# Engineering trap dynamics in Ba_2_ZnSi_2_O_7_ phosphors: a study on rare-earth co-doping for efficient TL dosimetry

**DOI:** 10.1039/d6ra07885k

**Published:** 2026-09-14

**Authors:** Tejas, Naregundi Karunakara, Nagaraj Kamath, Sudha D. Kamath

**Affiliations:** a Manipal Institute of Technology, Manipal Academy of Higher Education Manipal Karnataka India sudha.kamath@manipal.edu; b Center for Application of Radioisotopes and Radiation Technology (CARRT), Mangalore University Mangalagangothri Mangalore Karnataka India; c Center for Advanced Research in Environmental Radioactivity (CARER), Mangalore University Mangalagangothri Mangalore Karnataka India

## Abstract

The present study investigates the thermoluminescence properties of Ba_2_ZnSi_2_O_7_ phosphors co-doped with rare-earth ions (Dy^3+^, Sm^3+^, and Tb^3+^) for radiation dosimetry applications. The phosphors were synthesized with varying dopant concentrations, and their TL glow curves were recorded under γ-irradiation over a wide dose range. The glow curves exhibited well-defined peaks, indicating the presence of discrete trapping centers. The TL intensity showed a strong dependence on dopant concentration, with optimal compositions displaying maximum emission, while higher concentrations led to concentration quenching. Dose–response studies revealed a linear increase in TL intensity within specific dose regions, followed by saturation at higher doses due to trap filling. Sensitivity and minimum detectable dose were evaluated, demonstrating that the phosphors possess good detection capability across low to high dose ranges. The trapping parameters, including activation energy, frequency factor, and trap lifetime, were determined using Computerized Glow Curve Deconvolution, Chen's peak shape method, and the initial rise method. The calculated activation energies ∼1–1.8 eV indicate the presence of stable deep traps. The consistency among different analytical methods confirms the reliability of the results. Overall, the study highlights that Ba_2_ZnSi_2_O_7_ based phosphors are promising materials for radiation dosimetry, with tuneable properties suitable for radiation detection applications.

## Abstract 2

Rare earth doped phosphors for gamma irradiated TL applications.

## Graphical abstract

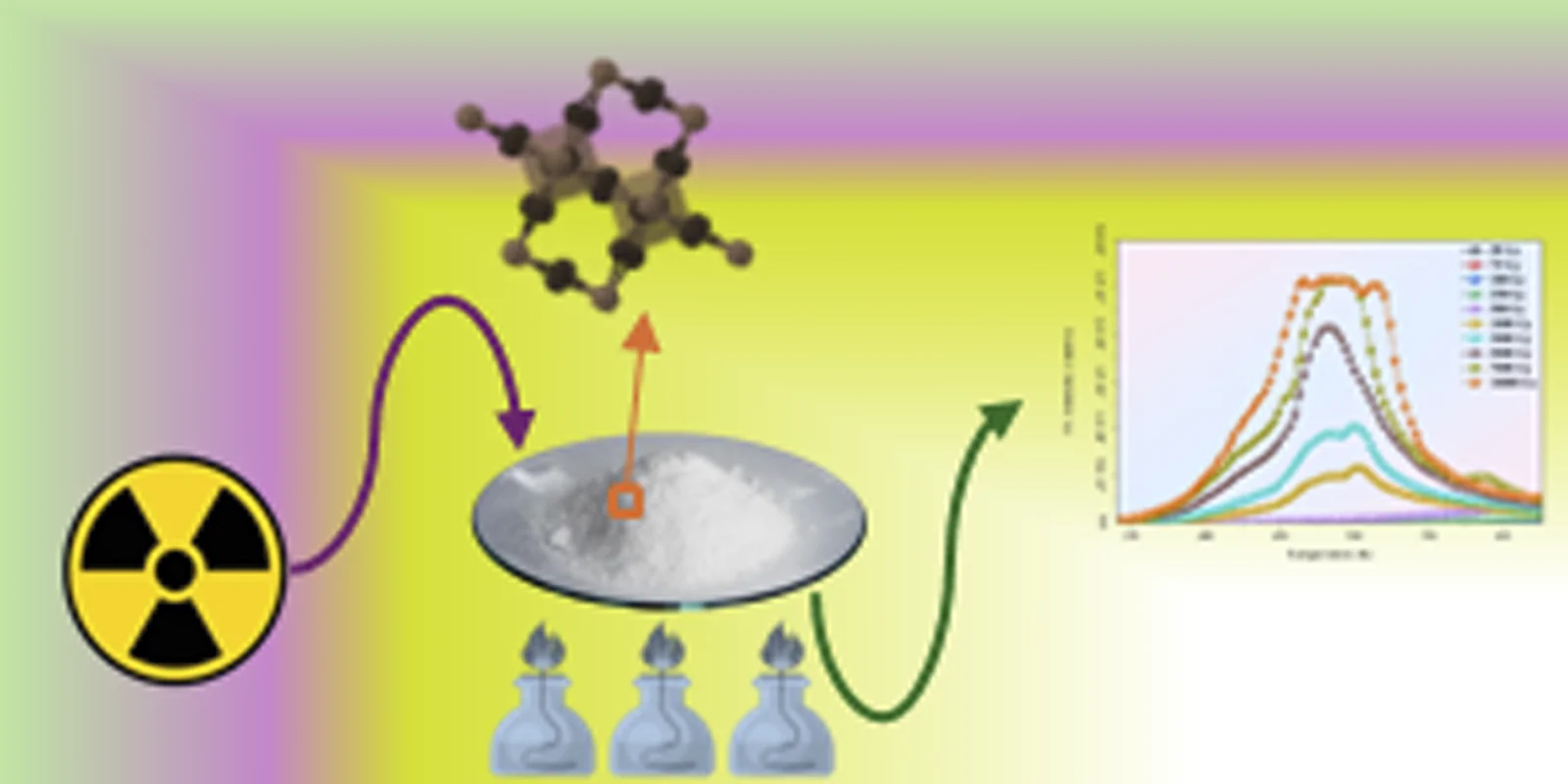

## 1. Introduction

Thermoluminescence has remained an active area of research for several decades because of its strong relevance to radiation dosimetry and defect studies in insulating and semiconducting materials.^1–3^ The TL phenomenon occurs when a material, after being exposed to ionizing radiation, emits light upon controlled heating. This emission results from the release of charge carriers that were previously trapped at defect-related energy levels within the band gap and their subsequent radiative recombination.^4^ The nature, distribution, and stability of these trapping and recombination centers largely determine the TL characteristics of a material. When the emitted intensity is recorded as a function of temperature, a characteristic glow curve is obtained. Each glow peak corresponds to a specific trapping level, and its position and shape provide insight into important trapping parameters such as activation energy (*E*), frequency factor (*s*), order of kinetics (*b*), and peak temperature (*T*_m_).^5,6^ These parameters govern the thermal stability of trapped carriers, re-trapping probability during heating, and ultimately the dosimetric performance of the phosphor. Techniques such as the initial rise method, Chen's peak shape analysis, and Computerized Glow Curve Deconvolution (CGCD) are commonly employed to evaluate these kinetic parameters from TL glow curves.^7,8^

Over the years, several materials have been investigated as thermoluminescent dosimeters (TLDs). Among them, Lithium fluoride-based phosphors have gained widespread acceptance due to their near tissue equivalence and reliable dose response.^9^ Other systems such as CaSO_4_: Dy and Al_2_O_3_: Si, Ti have also shown promising dosimetric characteristics.^10–13^ Despite their success, the continuous demand for materials with higher sensitivity, improved thermal stability, low fading, and better reusability has encouraged the exploration of alternative host lattices.^14^ Silicate-based materials are particularly attractive in this regard because of their chemical stability, wide band gap, and structural flexibility for accommodating rare-earth dopants. In this context, Barium zinc silicate (Ba_2_ZnSi_2_O_7_) emerges as a promising host owing to its stable crystal structure and ability to incorporate trivalent rare-earth ions without significant structural distortion.^15^ Rare-earth ions such as Dy^3+^, Sm^3+^, and Tb^3+^ are known to introduce effective trapping and recombination centers, and their interaction in co-doped systems can modify the trap distribution and enhance TL response through possible synergistic effects.^16–19^ In our previous work, we have already discussed thermoluminescence characteristics of singly doped Dy^3+^, Sm^3+^, Tb^3+^ in Ba_2_ZnSi_2_O_7_ phosphors in detail.^20^

In our earlier work, the structural, morphological, and basic luminescence properties of Ba_2_ZnSi_2_O_7_ co-doped with Dy^3+^/Sm^3+^ (ref. 21) and Tb^3+^/Sm^3+^ (ref. 22) were systematically examined, and the optimum dopant concentrations for maximum PL intensity were identified. Phase purity and successful incorporation of dopant ions into the host lattice were confirmed through structural analysis. The present study builds upon those findings and focuses on a comprehensive thermoluminescence investigation of the optimized Ba_2_ZnSi_2_O_7_:Dy^3+^/Sm^3+^, Ba_2_ZnSi_2_O_7_:Sm^3+^/Tb^3+^, and Ba_2_ZnSi_2_O_7_:Tb^3+^/Sm^3+^ phosphor systems. Detailed analysis of glow curve behaviour, trapping parameters, dose response characteristics, and sensitivity has been carried out to assess their suitability for radiation dosimetry applications. A comparative evaluation of the three co-doped series is presented to understand the influence of different rare-earth combinations on trap dynamics and overall TL performance. Through this study, an effort has been made to identify a stable and efficient thermoluminescent material based on the Ba_2_ZnSi_2_O_7_ host lattice for potential dosimetric use.

## 2. Experimental and methodology

The phosphor samples were synthesized by a conventional solid-state reaction route. High-purity starting materials were used throughout the preparation, including dysprosium oxide (Dy_2_O_3_, 99.99%, Loba), samarium oxide (Sm_2_O_3_, 99.99%, Loba), terbium oxide (Tb_4_O_7_, 99.99%, MolyChem), barium carbonate (BaCO_3_, 99.99%, Alfa Aesar), zinc oxide (ZnO, 99.99%, Alfa Aesar), and silicon dioxide (SiO_2_, 99.9%, MolyChem). The required amounts of raw materials were calculated according to the stoichiometric composition for Dy^3+^, Sm^3+^, and Tb^3+^ doped Ba_2_ZnSi_2_O_7_ phosphors. The weighed powders were thoroughly homogenized by manual grinding in an agate mortar for approximately one hour to ensure uniform mixing. The homogeneous mixture was then transferred into an alumina crucible and subjected to heat treatment in air. The furnace temperature was raised to 1200 °C at a controlled heating rate of 4 °C min^−1^ and maintained at this temperature for 6 hours to facilitate complete solid-state reaction. After sintering, the samples were allowed to cool gradually to room temperature inside the furnace at the same rate of 4 °C min^−1^. The obtained bulk product was subsequently reground into fine powder form for further characterization, following the procedure reported in our earlier studies.^23^

Thermoluminescence studies were carried out on the synthesized phosphor powders after exposing them to gamma radiation using a Gamma Chamber-5000 fitted with a ^60^Co source. The irradiation dose was systematically varied between 5 Gy and 10 KGy to investigate the dose-dependent TL characteristics. For each measurement, nearly 10 mg of the sample was taken. Heating rate was fixed to 2.85 K s^−1^ with temperature range from 300–650 K. The TL glow curves were obtained using a TLD Reader 1009I (Nucleonix, India), with the samples heated at a uniform and controlled rate. To better understand the trapping mechanisms, the resulting glow curves were subjected to computerized deconvolution, enabling separation of overlapping peaks. Further, kinetic parameters were extracted from the resolved peaks, and the trap depths were estimated using suitable graphical analysis techniques.

## 3. Results and discussion

### 3.1. Thermoluminescence studies of Ba_2_ZnSi_2_O_7_: 1.5 mol% Dy^3+^, *x* Sm^3+^ (*x* = 0.2, 0.5, 1, 2, 3 mol%) phosphors

To determine the most suitable rare-earth doping concentration, different amounts of Dy_2_O_3_ and Sm_2_O_3_ were introduced during the synthesis stage. Dy^3+^ ions were already optimized through PL in our previous work.^24^ For thermoluminescence measurements, all samples were prepared with a mass of 15 mg. Each sample was irradiated with a radiation dose of 500 Gy, and the TL glow curves were recorded using a heating rate of 2.85 K s^−1^. The resulting TL glow curves are presented in Fig. 1. The mechanism of the thermoluminescence response of the Dy^3+^/Sm^3+^ co-doped Ba_2_ZnSi_2_O_7_ phosphors is interpreted using a common defect-assisted trapping mechanism applicable to all investigated compositions. The substitution of trivalent Dy^3+^ and Sm^3+^ ions at Ba^2+^ lattice sites introduces charge imbalance, which is compensated by the formation of intrinsic lattice defects, primarily barium vacancies and oxygen-related defects. These defects create localized energy levels within the band gap that act as charge trapping centers during gamma irradiation. Gamma irradiation generates electron-hole pairs in the host lattice, and a fraction of these carriers is trapped at defect levels associated with the rare-earth-induced lattice imperfections. Upon thermal stimulation, the trapped carriers are released and recombine at luminescent centers, producing the observed thermoluminescence glow peaks. The calculated activation energies correspond to thermally stable traps suitable for gamma-ray thermoluminescence measurements. Rare-earth co-doping also modifies the local crystal field around the dopant ions because of the ionic size and charge mismatch between Ba^2+^ and the rare-earth ions. The resulting lattice distortion influences the formation energy and distribution of trapping centers, leading to variations in thermoluminescence intensity and trap depth among different compositions. Therefore, the differences observed across the doping series are attributed to changes in the concentration and distribution of these defect-related traps rather than to different trapping mechanisms.

**Fig. 1:**
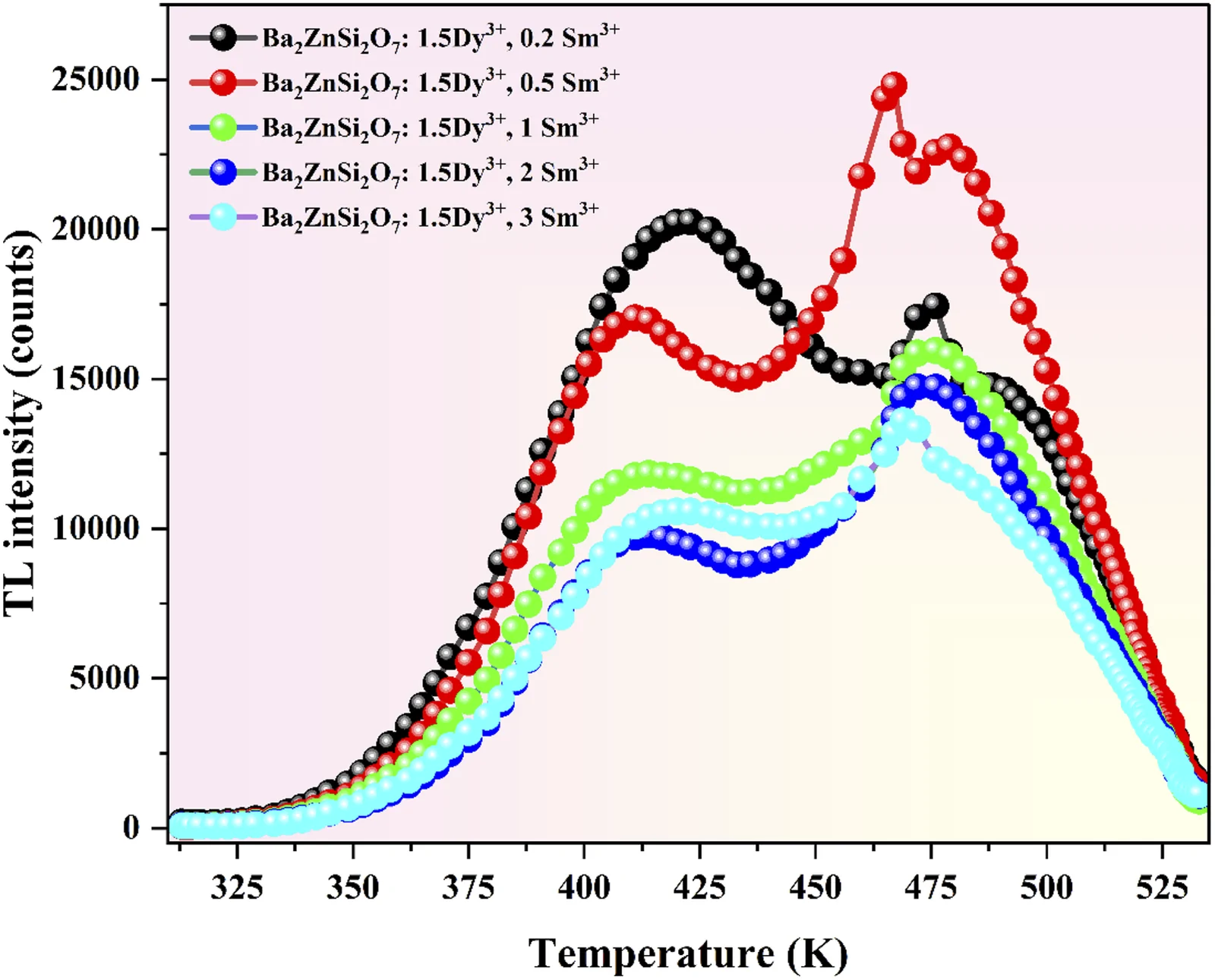
TL glow curves for 500 Gy γ-doses of Ba_2_ZnSi_2_O_7_:1.5 mol% Dy^3+^, *x*Sm^3+^ phosphors (*x* = 0.2, 0.5, 1, 2, and 3 mol%).

As shown in Fig. 1, the TL glow curve of the samples exhibits two main peaks located at approximately 410 K and 475 K. When the concentration of Sm^3+^ ions was gradually increased from 0.2 to 3 mol%, noticeable changes in the TL glow curves were observed. The maximum TL peak observed at 0.5 mol% Sm^3+^ concentration which is used for different doses. Fig. 2 shows the optimum concentration doping rare earth ions for different doses. TL integral intensity initially increases with increasing Sm^3+^ concentration and then saturates after 10KGy. Among the investigated concentrations, the maximum TL integral intensity was obtained at a 10KGy radiation dose at 0.5 mol% Sm^3+^.

**Fig. 2:**
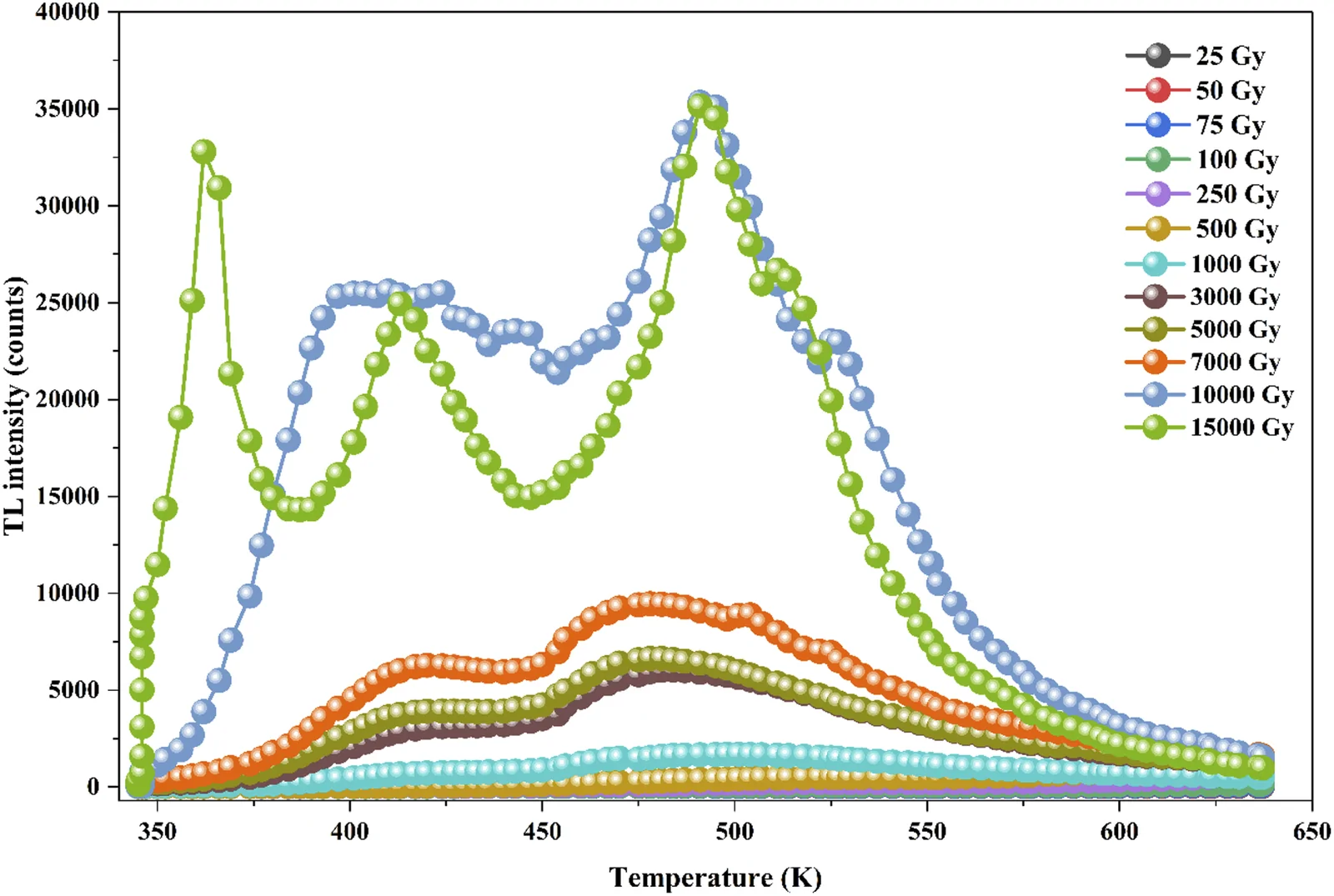
Thermoluminescence glow curves of Ba_2_ZnSi_2_O_7_ phosphor co-doped with 1.5 mol% Dy^3+^ and 0.5 mol% Sm^3+^ recorded at various γ irradiation doses.

The TL integrated intensity *vs.* dosage graph in Fig. 3 had a similar pattern. TL integral intensity, which is calculated by adding the area under the glow curve, shows the entire amount of light released by the sample as it is heated over a predetermined temperature range. For more clarity, Fig. 3 inset offers a closer look at the lower dosage region.

**Fig. 3:**
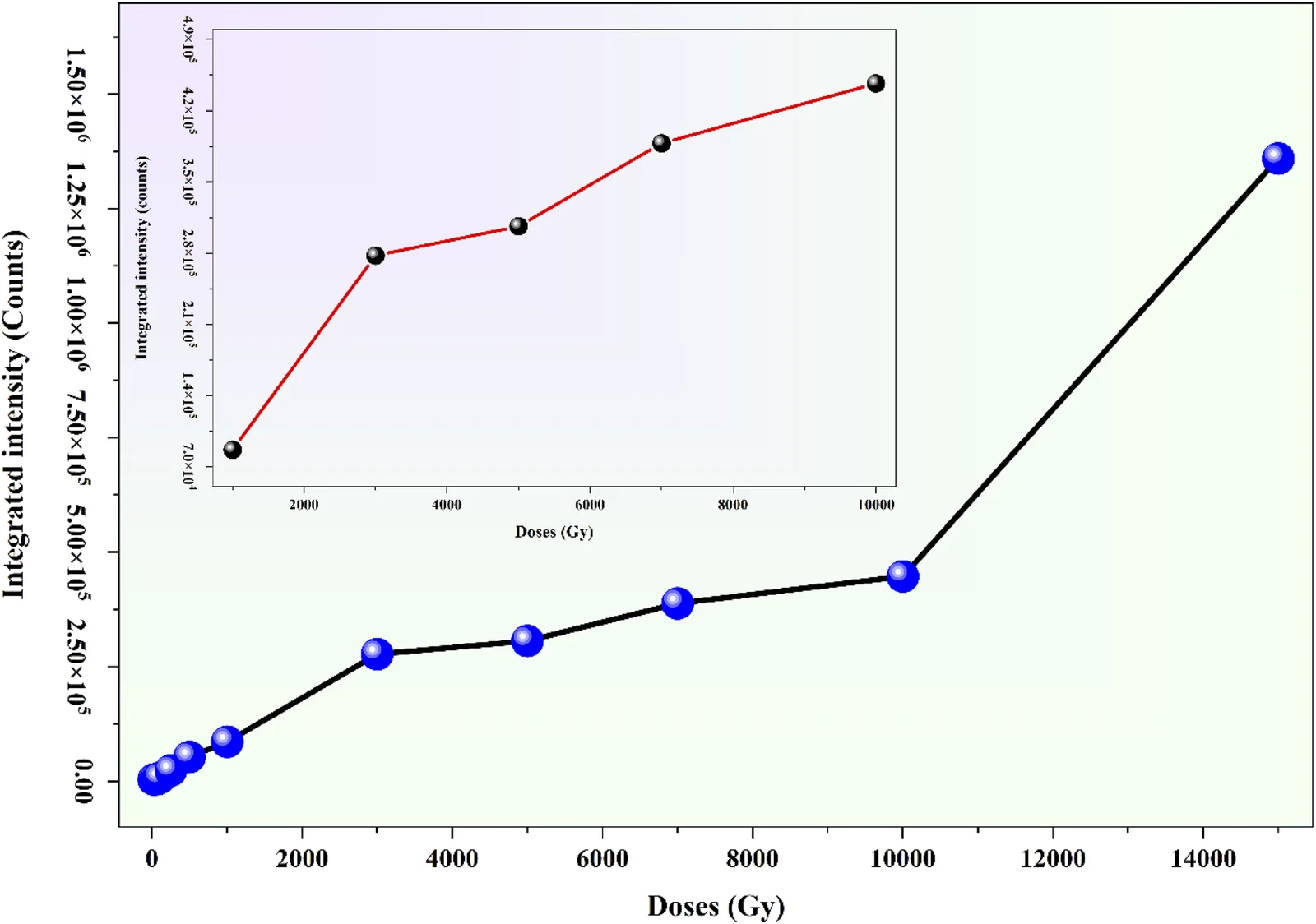
TL intensity variation with doses.

#### 3.1.1. Sensitivity

The variation in TL response shows that the phosphor maintains a linear relationship with radiation dose up to about 10 KGy. Beyond this point, the response begins to deviate and displays a sub-linear trend. Fig. 4 presents the dose–response behaviour of the Ba_2_ZnSi_2_O_7_:1.5 mol% Dy^3+^, 0.5 mol% Sm^3+^ phosphors in the dose range of 25–15000 Gy. A linear fit applied to the data between 3000 and 10 000 Gy gives a high correlation coefficient of 0.95, indicating a strong and reliable linear dependence of TL intensity on the applied dose. However, for doses higher than 10 KGy, the deviation from linearity becomes noticeable, as seen in Fig. 4. The observed sub-linear response can be understood based on the explanation suggested by J. L. Lawless *et al.*,^25^ which attributes this behaviour to the competition between recombination centers and trapping centers. As the irradiation dose increases, additional trapping sites may be generated in the material instead of the already existing recombination centers being fully occupied. These newly formed traps capture the charge carriers and reduce the probability of recombination, which in turn lowers the emitted TL intensity compared to the expected value. Because of this effect, the TL output increases at a slower rate, resulting in sub-linear behaviour. Therefore, 10 KGy, which represents the upper limit of the linear response region, was selected as the optimum dose for further analysis. Another important parameter that determines the performance of a TLD material is its thermoluminescence sensitivity, especially for low dose monitoring. TL sensitivity is generally defined as the emitted TL intensity per unit mass of the sample for a given absorbed radiation dose. This parameter is influenced by several experimental factors, including the heating rate used during TL readout, the characteristics of the measurement system, the nature and preparation of the irradiated sample, and the type of radiation involved. For the present phosphor composition, Ba_2_ZnSi_2_O_7_:1.5 mol% Dy^3+^, 0.5 mol% Sm^3+^, the calculated TL sensitivity was found to be 2547 counts g^−1^ Gy^−1^, indicating a strong luminescent response to the absorbed radiation dose as shown in inset Fig. 4.

**Fig. 4:**
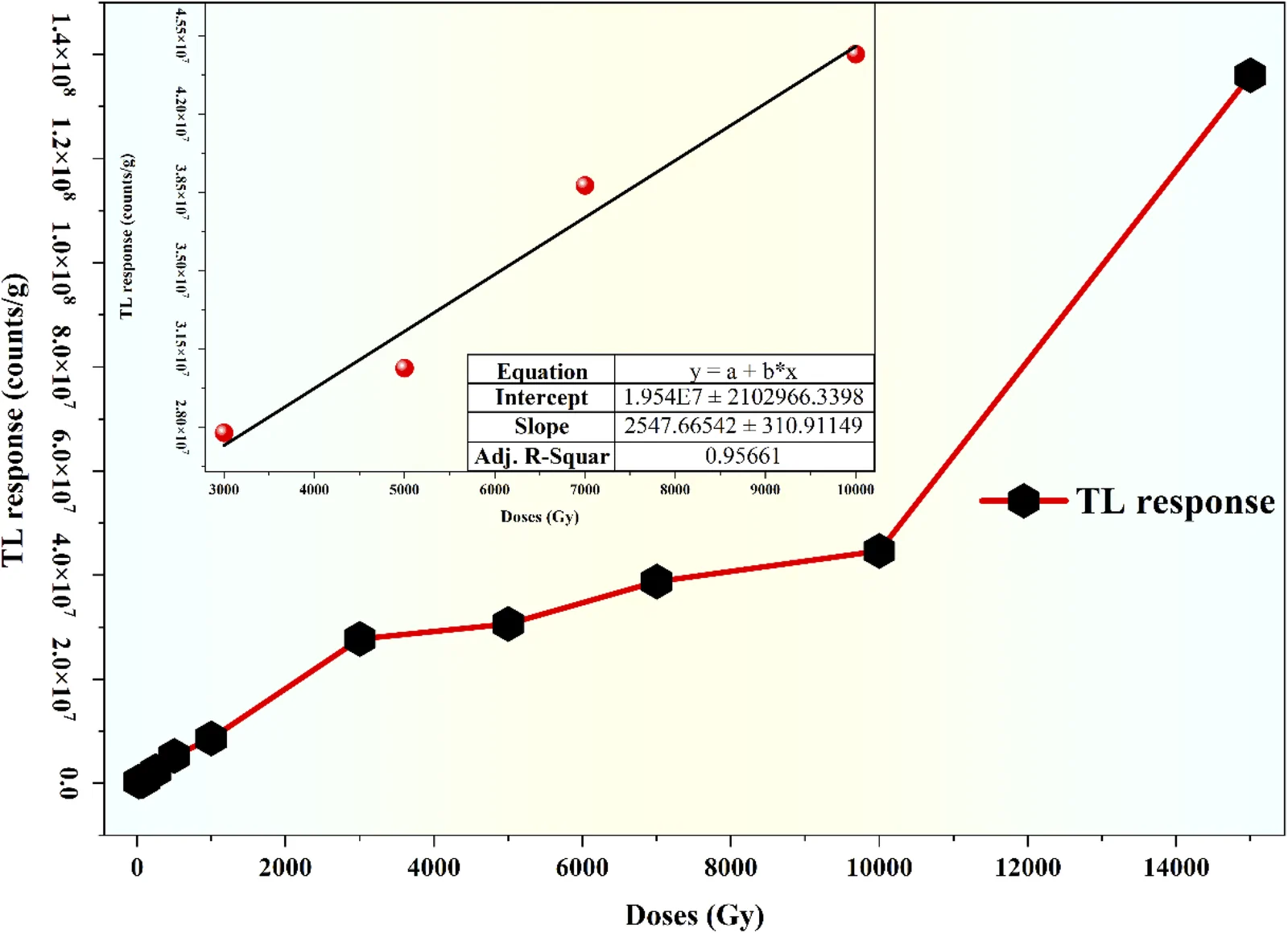
TL response plot for Ba_2_ZnSi_2_O_7_:1.5 mol% Dy^3+^, 0.5 mol% Sm^3+^ phosphors.

#### 3.1.2. Minimum detectable dose

MDD of the thermoluminescent dosimeter, background TL glow curves were recorded for three unirradiated samples of Ba_2_ZnSi_2_O_7_:1.5 mol% Dy^3+^, 0.5 mol% Sm^3+^ phosphor. The integrated TL intensities obtained from these measurements were used to determine the standard deviation of the background signal (*σ*_b_). The minimum detectable dose defines the lowest dose that can be distinguished from the background signal and should not be confused with the linear dose–response region. The TL dose–response exhibits the characteristic trap-filling behaviour of thermoluminescent phosphors, consisting of an initial non-linear region (25–3000 Gy), a linear region (3000–10000 Gy), and an eventual saturation region at doses beyond the investigated range. The MDD was then calculated using the relation given in ref. 26 and 27,

1 MDD = 3 × *σ*_b_ × *F*

where *F* represents the calibration factor, which is the inverse of the sensitivity of the phosphor. The calculated minimum detectable dose for Ba_2_ZnSi_2_O_7_:1.5 mol% Dy^3+^, 0.5 mol% Sm^3+^ phosphor was 440.16 mGy, suggesting that this material can effectively detect high levels of radiation and may be useful for high-dose dosimetry applications.

#### 3.1.3. Kinetic parameters

##### 3.1.3.1. Computerized glow curve deconvolution method

Thermoluminescence occurs when charge carriers that are trapped in defect states are released during heating. The TL mechanism may vary from one host lattice to another because the type and origin of trapping centers are different for each material. In the prepared phosphors, when RE^3+^ ions substitute Ba^2+^ sites in the host lattice, charge compensation leads to the formation of Ba^2+^ vacancies (V_Ba_). These vacancies can act as hole trapping centers in the phosphor. In addition, some reports suggest the presence of oxygen vacancies (V_O_) that behave as electron traps.

When the material is exposed to γ-radiation, electrons and holes are generated. The holes migrate through the lattice and can become self-trapped at nearby sites due to electron–phonon interactions. During subsequent heating, these trapped carriers are released and recombine at RE^3+^ luminescent centers, which results in the emission of light. The analysis of trap parameters helps in understanding the defect levels present in the material. Important parameters such as the frequency factor (*s*), trap depth (*E*), lifetime (*t*), and kinetic order (*b*) provide useful insight into the potential of the phosphor for radiation dosimetry. In the present work, these parameters were evaluated using the computerized glow curve deconvolution (CGCD) technique. The experimental TL glow curve was fitted with a theoretical curve generated using Kitti's general-order kinetic equation given in eqn (2).^28^ Initially, approximate trap positions were identified, and then the values of trap depth (*E*) and kinetic order (*b*) were refined through an iterative fitting process. The iterations were continued until a good agreement between the experimental and theoretical curves was achieved, corresponding to the minimum value of the figure of merit (FOM) as given in eqn (3).^28^ The GCD computerized fitting is done using MS Excel software provided by M. W. Din published in Zenodo website.^29^2
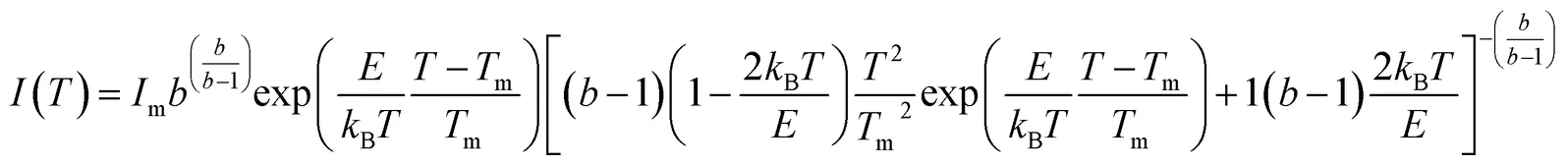
3
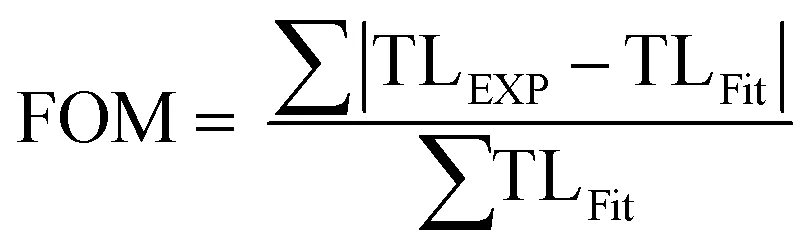
Here, *I*(*T*) denotes TL intensity measured at a specific temperature *T*, while *b* represents the kinetic order associated with the glow peak. The parameter *I*_m_ indicates the peak TL intensity, *k*_B_ refers to the Boltzmann constant, and *T*_m_ is temperature corresponding to the maximum intensity of the glow peak. *T* indicates absolute temperature, and *E* represents activation energy or trap depth. The experimental glow curve was deconvoluted in such a way that the fitting error, expressed in terms of the figure of merit (FOM), remained below 5%.^30^ Furthermore, eqn (4) and (5) were employed to calculate the trap lifetime and the frequency factor (*s*) of the trapping centers.^31^4
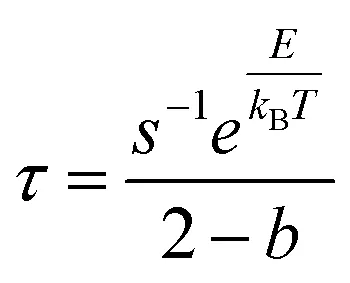
5
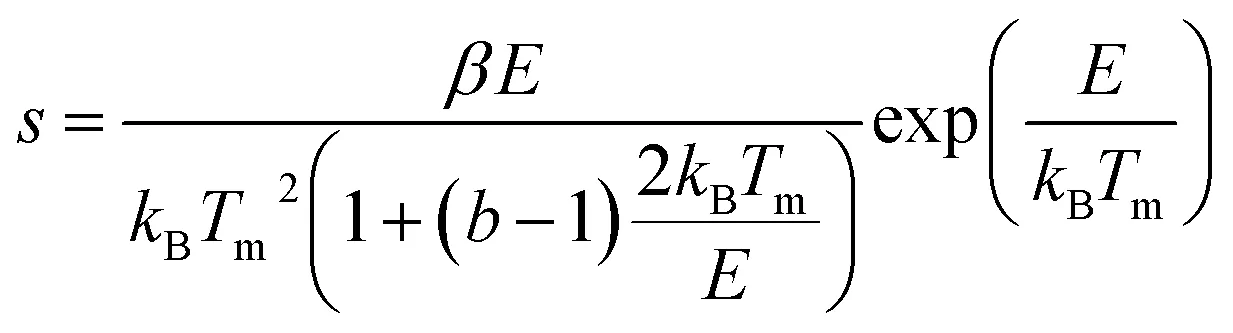
Here, *τ* represents the lifetime of the trap, while *s* denotes the frequency factor associated with the trapping center. *T*_m_ refers to the temperature at which the TL peak reaches its maximum intensity, and *T* is the storage temperature, taken as 300 K. *β* indicates the heating rate used during the TL measurement (2.85 K s^−1^), and *b* corresponds to the order of kinetics of the glow peak.

Deconvoluted TL curves of Ba_2_ZnSi_2_O_7_:1.5 mol% Dy^3+^, 0.5 mol% Sm^3+^ phosphor for the 3000–10 000 Gy dose are plotted in Fig. 5, and the trap parameters are given in Table 1. Each TL glow curve corresponding to different irradiation doses was deconvoluted into three individual peaks, indicating the presence of three distinct trapping centers in the samples. The traps appearing at lower temperatures are relatively shallow, whereas those observed at higher temperatures correspond to deeper traps. From the analysis of the calculated trap parameters, it is evident that the trap temperatures shift towards higher values with an increase in dose, suggesting the formation of deeper trapping levels. Among these, the traps located in the temperature range of 483–503 K exhibit the highest intensity, indicating a larger concentration of deep traps in the samples. As a result, a greater amount of thermal energy is required to release the trapped charge carriers from these levels. This behaviour supports the potential applicability of the prepared phosphors for γ-ray dosimetry. Furthermore, deeper traps with higher activation energy are generally more stable and tend to exhibit lower fading over time compared to shallow traps. The calculated trap lifetimes are theoretical estimates derived from activation energy and frequency factor at room temperature. Extremely large lifetime values indicate highly stable deep traps with low thermal release probability and should be interpreted as a measure of trap stability rather than actual experimental storage times.

**Fig. 5:**
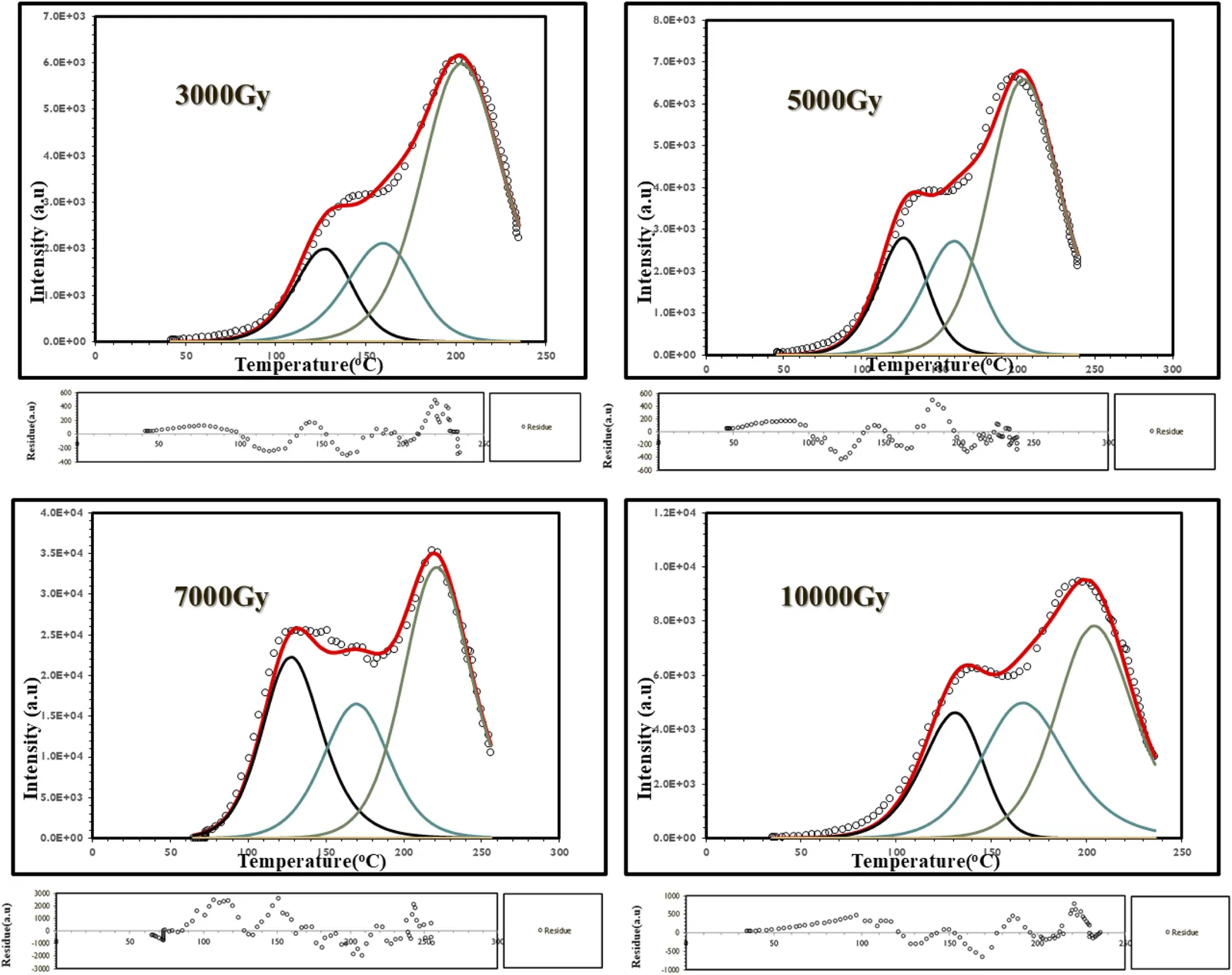
Deconvoluted glow curve of Ba_2_ZnSi_2_O_7_:1.5 mol% Dy^3+^, 0.5 mol% Sm^3+^ phosphor for different doses.

**Table 1:** Determination of trap energies in Ba_2_ZnSi_2_O_7_:1.5 mol% Dy^3+^, 0.5 mol% Sm^3+^ phosphor using multiple evaluation techniques

Doses (Gy)	Peak number	*T*_m_ (K)	CGCD					Chen's		Initial rise
			*E* (eV)	*b*	FF (s^−1^)	Lifetime (days)	FOM (%)	*E* (eV)	FF (s^−1^)	*E* (eV)
3000	1	401.1	1.00	1.54	7.92 × 10^11^	2.32	4.98	0.97	2.52 × 10^11^	1.18
	2	432.8	1.00	1.45	7.69 × 10^10^	23.92		1.02	1.29 × 10^11^	0.98
	3	476.5	1.20	1.99	8.32 × 10^11^	6093.83		1.17	3.85 × 10^11^	1.01
5000	1	400	1.06	1.53	4.80 × 10^12^	4.12	4.78	1.03	2.21 × 10^12^	1.01
	2	432.2	1.07	1.46	5.39 × 10^11^	54.60		1.02	1.46 × 10^11^	0.98
	3	477.5	1.20	1.99	7.79 × 10^11^	6508.43		1.15	2.17 × 10^11^	1.18
7000	1	400.8	1.01	1.99	9.99 × 10^11^	2.73	4.96	1.01	1.00 × 10^12^	0.89
	2	442.7	1.01	1.66	5.18 × 10^10^	52.77		1.00	4.39 × 10^11^	0.95
	3	495	1.34	1.99	8.00 × 10^12^	162 219.32		1.52	5.33 × 10^14^	1.32
10 000	1	404.2	1.00	1.3	5.92 × 10^11^	3.10	4.99	0.97	2.57 × 10^11^	0.96
	2	439.4	1.04	1.98	1.40 × 10^11^	64.07		1.05	1.98 × 10^11^	1.01
	3	477	1.35	1.99	3.40 × 10^11^	56 718.71		1.33	2.14 × 10^13^	1.33

##### 3.1.3.2. Chen's peak shape method

The trapping parameters are also calculated through Chen's peak shape method.^32,33^Eqn (6)–(8) have been used to extract the kinetic parameters:

6 *τ* = *T*_m_ − *T*_1_

7 *δ* = *T*_2_ − *T*_m_

8 *ω* = *T*_2_ − *T*_1_

Geometrical factor is given by,^34^9
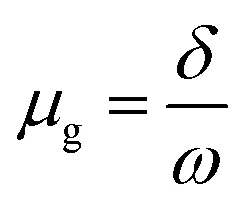


In this context, *T*_m_ represents temperature at which the TL glow peak reaches its maximum intensity. The parameters *T*_1_ and *T*_2_ correspond to the temperatures at half of the peak intensity on the lower and higher temperature sides of *T*_m_, respectively. The symmetry factor (*µ*_g_) describes the geometrical shape of the glow peak, while *ω* denotes the total half-width of the peak. The terms *τ* and *δ* represent the half-widths on the low-temperature and high-temperature sides of the peak, respectively. The value of the symmetry factor helps determine the order of kinetics governing the glow peak. When *µ*_g_ ≈ 0.42, the peak generally follows first-order kinetics, whereas a value close to 0.52 indicates second-order kinetics. Based on these parameters, the activation energy (*E*_α_) of the traps can be estimated using the following relation,^35,36^

10 *C*_τ_ = 1.51 + 3(*µ*_g_ − 0.42); *b*_τ_ = 1.58 + 4.2(*µ*_g_ − 0.42)

11 *C*_δ_ = 0.976 + 7.3(*µ*_g_ − 0.42); *b*_δ_ = 0

12 *C*_ω_ = 2.52 + 10.2(*µ*_g_ − 0.42); *b*_ω_ = 1

13
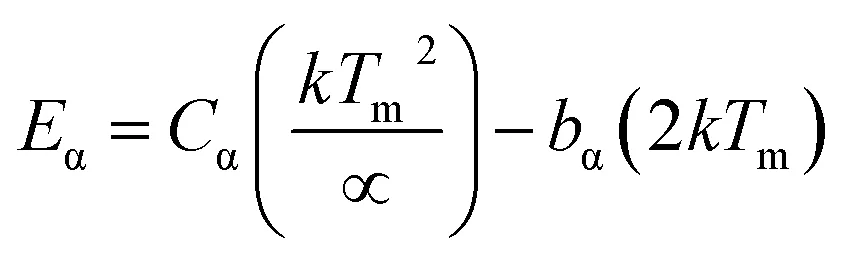
Here, *α* represents the peak shape parameters *τ*, *δ*, and *ω*. The activation energy of the trap is determined by calculating *E*_τ_, *E*_δ_, and *E*_ω_ using these parameters, and the average of these three values is taken as the final trap activation energy. The kinetic parameters of the glow peaks were evaluated using Chen's peak shape method, and the calculated values are summarized in Table 1. The trapping and recombination processes mainly follow second-order kinetics, since the values of the symmetry factor *µ*_g_ for most of the traps are close to 0.52. The calculated activation energies are found to be nearly the same when determined using both methods. It is also observed that the trap lifetime increases with increasing doses. A similar trend is obtained for the trap lifetimes estimated through the CGCD technique, suggesting that charge carriers trapped in deeper energy levels remain stored for a longer period of time before being released.

##### 3.1.3.3. Initial rise method

The initial rise method, originally proposed by Garlick and Gibson,^37^ is based on examining the low-temperature portion of a TL glow peak up to a defined cut-off temperature (*T*_c_). In this region, the TL intensity is generally restricted to about 15% of the peak maximum (*I*_c_), and thus *T*_c_ corresponds to the temperature at which this ∼15% intensity level is reached. Within this initial segment, the population of trapped charge carriers can be assumed to remain nearly unchanged, as the likelihood of re-trapping is minimal at lower temperatures. A key benefit of this method is that it enables the evaluation of trap activation energy without requiring prior information about the kinetic order or frequency factor. This is because, in the initial rise region, the TL intensity exhibits an exponential relationship with both temperature and activation energy. Based on this principle, the activation energy can be calculated using the expression provided in eqn (14),^38^14
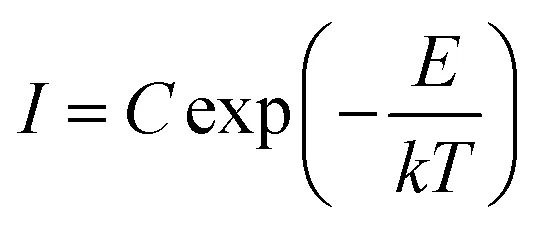


The activation energy can be evaluated by constructing a plot of ln(*I*) (for *I* < *I*_c_) *versus* 1/*kT*. This plot results in a straight line, and the slope of the line is equal to −*E*, from which the activation energy can be obtained. One advantage of this approach is that the value of *E* can be evaluated without prior knowledge of the frequency factor or the order of kinetics. Later, in 1960, Helfrich and Barner modified the original initial rise expression by incorporating general-order kinetics into the formulation. The modified relation is presented below as eqn (15),^39^15
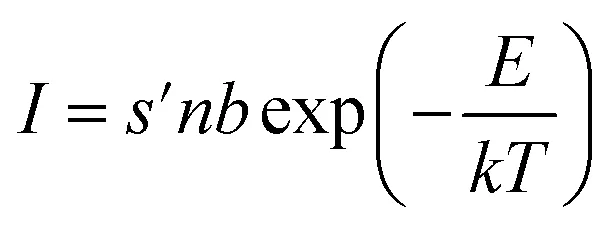
Here *s*′ represents the pre-exponential frequency factor, while *b* denotes the order of kinetics. According to the equation, several plots of ln (*I*) *versus* 1/*kT* are drawn for different assumed values of *b*. Among these plots, the line that shows the best linear behaviour is selected, and the corresponding value of *b* is considered as the appropriate kinetic order. The value of *n* can then be calculated using the specified relation in combination with the known heating rate *β*,^40^16
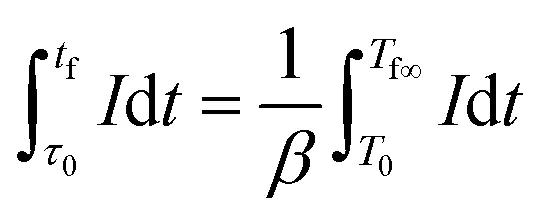


In this context, *I*d*t* represents the area of the initial rise region of the glow peak between a chosen starting temperature *T*_0_ and the final temperature *T*_f_. To accurately determine the activation energy of glow peaks in a TL phosphor, it is important to separate any overlapping peaks present in the glow curve. If the peaks overlap significantly, the initial rise (IR) method may not yield reliable activation energy values.^41^ Several techniques exist for separating composite peaks, one of which is thermal cleaning, where the sample is gradually heated to specific temperatures to remove carriers from different traps. However, this approach can sometimes remove part of the initial rise region of the peaks, which may affect the accuracy of the calculated activation energy. Therefore, computerized methods such as computerized glow curve deconvolution (CGCD) is commonly used to separate individual glow peaks more effectively.

In this case, three distinct TL peaks, observed at 400, 432 and 476 K, respectively. Although these peaks are not well separated, the glow curve was further deconvoluted using the CGCD technique to improve the accuracy of the analysis before applying the IR method. Plots of ln(*I*) *versus* 1/*kT* were constructed for all three peaks, corresponding to a cut-off intensity of 15% of the maximum TL intensity as shown in Fig. S1–S4. The resulting plots produced straight lines, from which the activation energies were obtained from the slopes. The calculated values were shown in Table 1. These values are in good agreement with those obtained using CGCD, Chen's peak shape method, indicating the reliability of the results. In general, the initial rise method can estimate activation energy with an uncertainty of approximately 5–10%.^42^

### 3.2. Thermoluminescence studies of Ba_2_ZnSi_2_O_7_: 0.4 mol% Sm^3+^, *y* Tb^3+^ (*y* = 1, 1.25, 1.5, 1.75, 2 mol%) phosphors

The thermoluminescence glow curves of Ba_2_ZnSi_2_O_7_:0.4 mol% Sm^3+^, yTb^3+^ (*y* = 1, 1.25, 1.5, 1.75, and 2 mol%) phosphors were measured from RT to 523 K at a heating rate of 2.85 K s^−1^, after irradiation using a Co_60_ gamma source. The recorded glow curves as shown in Fig. 6, reveal that the TL intensity initially increases with Tb^3+^ concentration and reaches a maximum at 1.25 mol%. Beyond this concentration, a reduction in intensity is observed, indicating concentration quenching. This behaviour is likely due to the decreased separation between dopant ions at higher concentrations, which enhances non-radiative interactions and disturbs the energy levels, ultimately lowering the TL output. The variation in TL intensity and trap parameters with Sm^3+^/Tb^3+^ concentration can be explained by the general defect-assisted trapping mechanism discussed in Section 3.1, where co-doping modifies the concentration and distribution of lattice trapping centers.

**Fig. 6:**
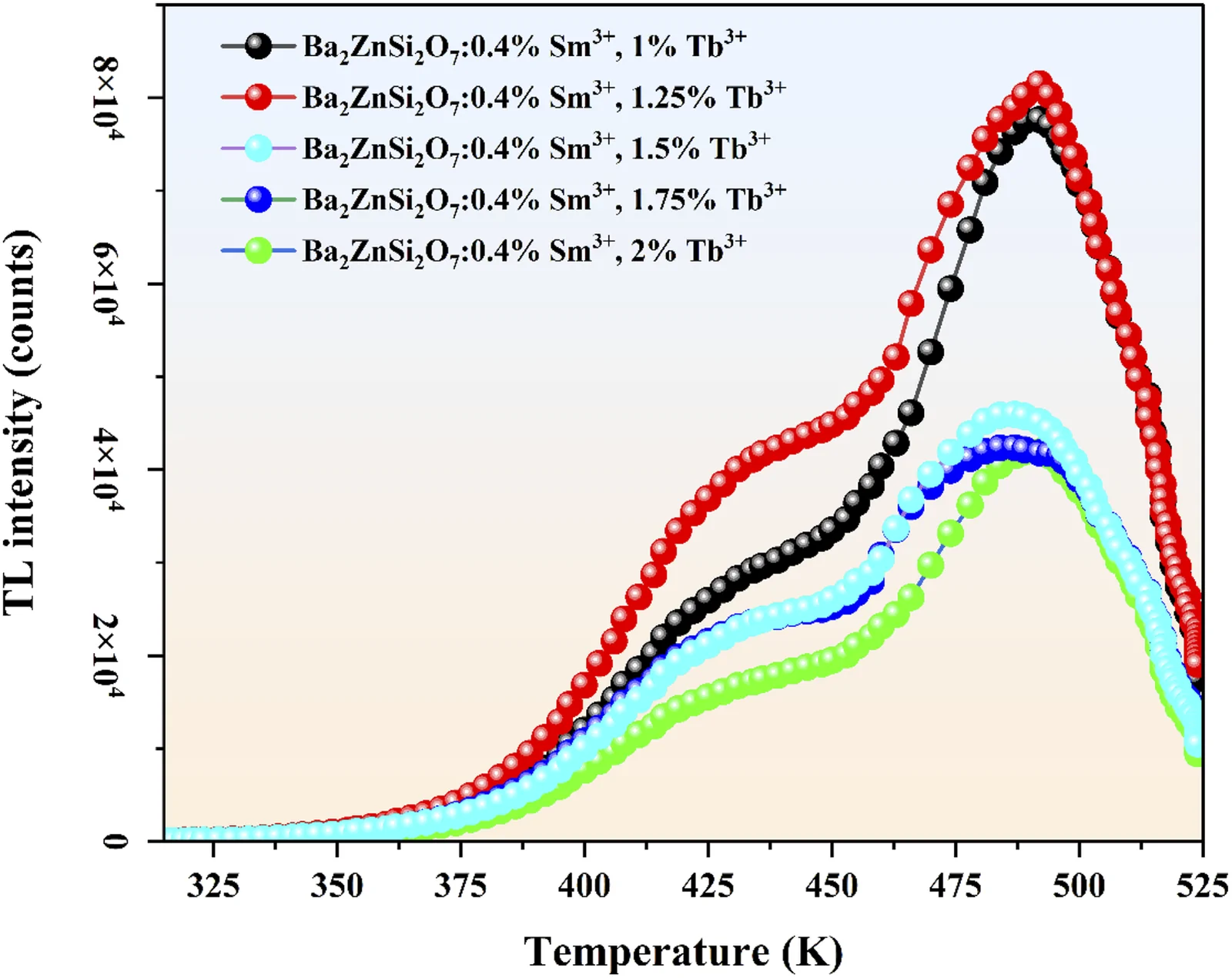
TL glow curves at 1 KGy dose for different concentration of Tb^3+^ ions in Ba_2_ZnSi_2_O_7_:0.4 mol% Sm^3+^.

Further investigation of the optimized composition Ba_2_ZnSi_2_O_7_:0.4 mol% Sm^3+^, 1.25 mol% Tb^3+^ under varying gamma doses from 25 Gy to 10 KGy as shown in Fig. 7, reflects that the shape and peak position of the glow curves remain nearly unchanged, while the TL intensity increases with dose. However, saturation of the TL signal is noticed beyond 10 KGy. The dose–response curve demonstrates a linear increase in TL intensity at mid lower doses, followed by a deviation from linearity at higher doses as shown in Fig. 8. As illustrated in Fig. 9, a clear linear response is maintained in the dose range of 500 Gy to 5000 Gy, indicating that defect generation is directly proportional to the absorbed radiation within this region.^27^

**Fig. 7:**
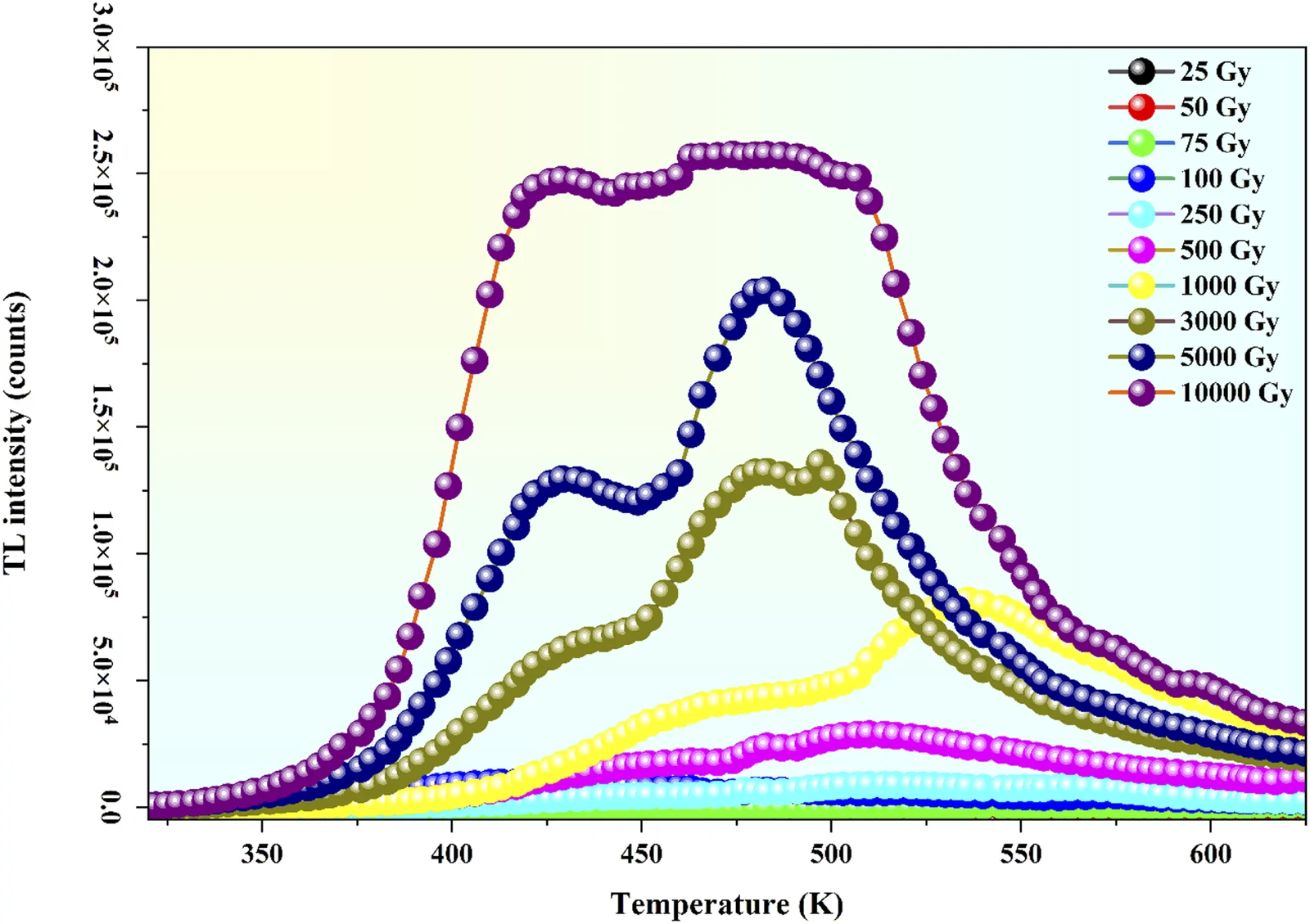
TL glow curve for Ba_2_ZnSi_2_O_7_:0.4 mol% Sm^3+^, 1.25 mol% Tb^3+^ for different doses.

**Fig. 8:**
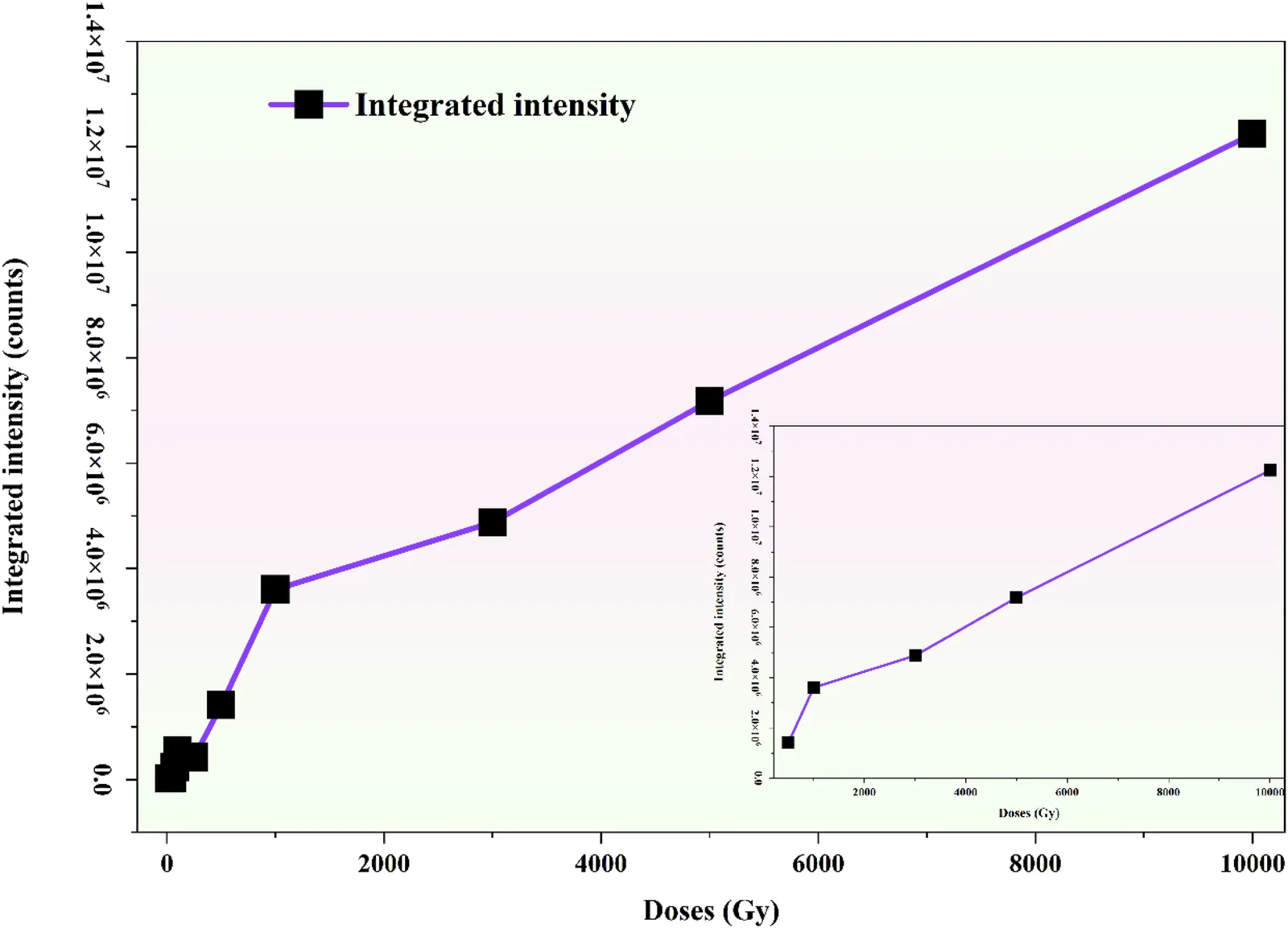
TL intensity variation for different doses.

**Fig. 9:**
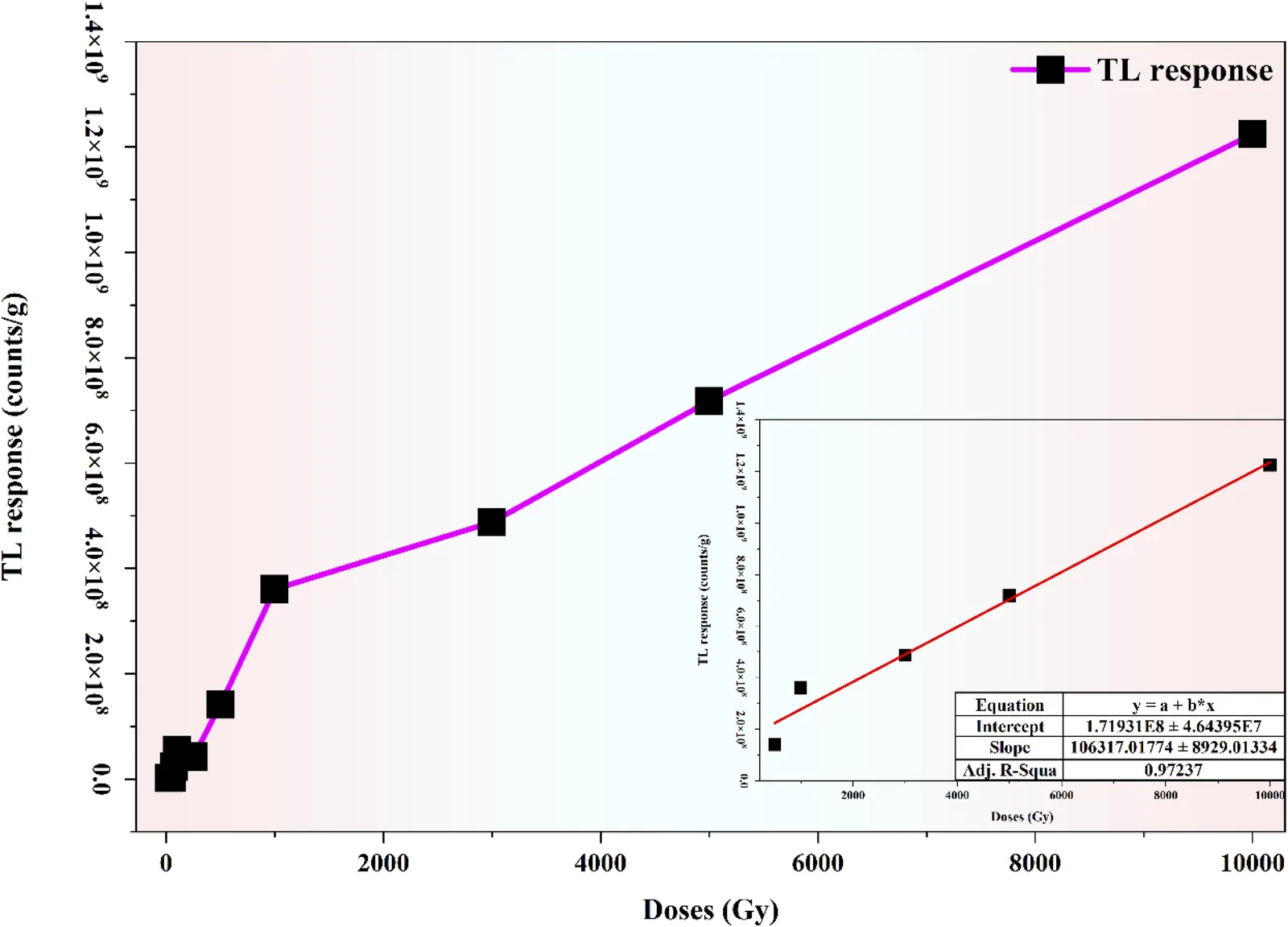
TL response plot for Ba_2_ZnSi_2_O_7_:0.4 mol% Sm^3+^, 1.25 mol% Tb^3+^.

#### 3.2.1. Sensitivity

TL sensitivity is an important parameter for materials used in personal and environmental radiation dosimetry. It is influenced by several factors, such as the heating rate employed during measurement, the detection system, the intrinsic properties of the sample, and the nature of the radiation source. In the case of Ba_2_ZnSi_2_O_7_:0.4 mol% Sm^3+^, 1.25 mol% Tb^3+^ phosphor, TL sensitivity was determined to be 106 317.01 counts g^−1^ Gy^−1^.

#### 3.2.2. Minimum detectable dose

MDD is estimated using the method suggested by Furetta *et al.*,^45^ based on the relation given in eqn (1). The calculation utilized slope of TL dose response curve presented in the inset of Fig. 9. From this analysis, MDD for Ba_2_ZnSi_2_O_7_:0.4 mol% Sm^3+^, 1.25 mol% Tb^3+^ was found to be about 134.73 mGy. This result suggests that the material is sensitive enough to measure moderate levels of radiation effectively.

#### 3.2.3. Kinetic parameters

##### 3.2.3.1. CGCD method

The emission occurs due to the efficient transfer of charge carriers from thermally stimulated trap to luminescence centers, where radiative recombination leads to light emission. The analysis of glow curves indicates that the TL behaviour of Ba_2_ZnSi_2_O_7_:0.4 mol% Sm^3+^, 1.25 mol% Tb^3+^ phosphor under γ irradiation suggests the presence of charge carrier re-trapping processes. Important trap-related parameters influencing TL emission were evaluated using the Computerized Glow Curve Deconvolution method.^43^Fig. 10 illustrates the fitting of glow curves at different irradiation doses for the optimized Tb^3+^ concentration in Ba_2_ZnSi_2_O_7_:Sm^3+^ phosphors. The corresponding kinetic parameters such as *b*, *E*, *τ*, and *s* are summarized in Table 2. The activation energy values were found to lie in the range of 1 to 1.56 eV, indicating that the luminescence originates from relatively deep trap levels.

**Fig. 10:**
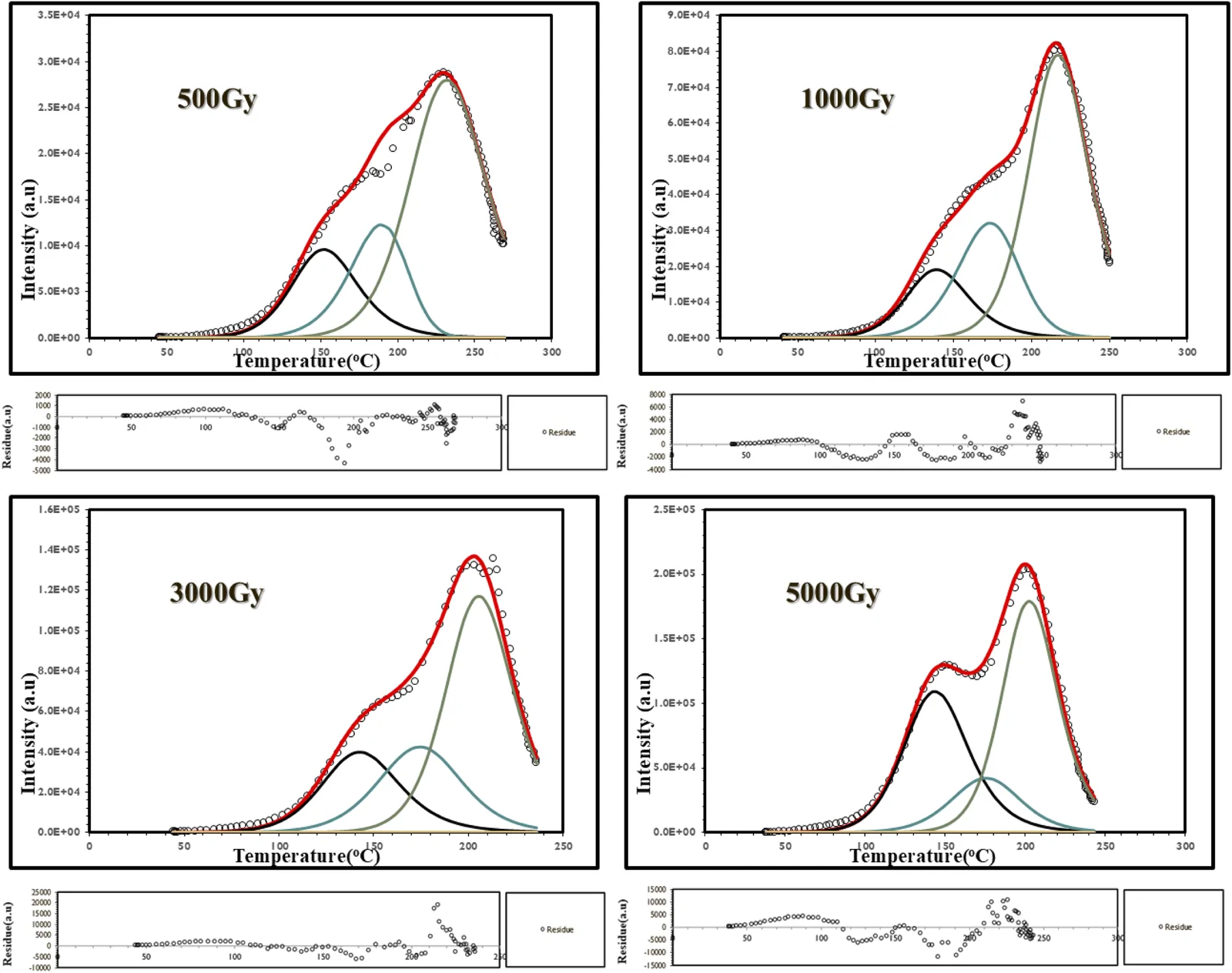
Deconvoluted TL glow curve for Ba_2_ZnSi_2_O_7_:0.4 mol% Sm^3+^, 1.25 mol% Tb^3+^ for linear doses.

**Table 2:** Comparison of trap energies for Ba_2_ZnSi_2_O_7_: 0.4 mol% Sm^3+^, 1.25 mol% Tb^3+^ obtained from various methods

Doses (Gy)	Peak number	*T*_m_ (K)	CGCD					Chen's		Initial rise
			*E* (eV)	*b*	FF (s^−1^)	Lifetime (days)	FOM (%)	*E* (eV)	FF (s^−1^)	*E* (eV)
500	1	425.17	1.01	1.98	1.64 × 10^11^	16.66	4.94	1.00	1.32 × 10^11^	1.00
	2	461.94	1.08	1.23	9.64 × 10^10^	453.71		1.17	1.10 × 10^12^	1.07
	3	505.31	1.22	1.99	2.24 × 10^11^	49 979.48		1.17	7.40 × 10^10^	1.21
1000	1	411.74	1.01	1.98	4.17 × 10^11^	6.55	4.96	1.05	1.38 × 10^12^	1.00
	2	446.40	1.02	1.43	5.38 × 10^10^	75.50		1.06	1.61 × 10^11^	1.01
	3	490.09	1.50	1.98	5.24 × 10^14^	1 399 796.91		1.47	7.12 × 10^15^	1.49
3000	1	416.01	1.01	1.97	3.22 × 10^11^	8.49	4.91	0.99	1.73 × 10^11^	0.99
	2	448.25	1.02	1.72	4.94 × 10^10^	82.23		0.98	1.56 × 10^10^	1.01
	3	479.41	1.56	1.99	5.77 × 10^15^	1 368 664.20		1.44	2.67 × 10^14^	1.55
5000	1	417.42	1.03	1.97	5.35 × 10^11^	11.28	4.93	0.96	7.18 × 10^10^	1.01
	2	448.26	1.04	1.72	7.94 × 10^10^	112.97		1.08	2.10 × 10^11^	1.03
	3	475.21	1.58	1.99	1.23 × 10^16^	1 417 728.52		1.67	1.24 × 10^17^	1.57

##### 3.2.3.2. Chen's peak shape method

In addition, Chen's peak shape method was employed for further analysis, following the formulations proposed by Kitis. This approach uses characteristic temperatures *T*_1_, *T*_m_, and *T*_2_ obtained from the glow curve to calculate shape parameters like *δ*, *ω*, and *τ*. These parameters, illustrated in Fig. S5–S8, are used to determine the symmetry factor (*µ*_g_), which was found to be in the range of 0.47–0.55. Based on these values, the kinetic process is identified as first-order in nature. Subsequently, trapping parameters were calculated using Kitis' equations. The average activation energy obtained through this method closely matches the values derived from the CGCD technique, as shown in Table 2. The strong agreement between both methods confirms the reliability and consistency of the experimental and theoretical analyses.

##### 3.2.3.3. Initial rise method

To find the trapping parameters using TL glow curves, the Garlick and Gibson approach is frequently utilized. It concentrates on the first rising part of the glow curve, when the inverse of temperature is shown against the natural logarithm of intensity. The slope of the straight-line fit in this area is then used to determine the activation energy. This method's applicability to all kinetic orders and lack of requirement for prior knowledge of the frequency factor are two of its main features. The first rising zones and the related linear fits for both samples are shown in Fig. S5–S8. Table 2 lists the computed activation energy.

### 3.3. Thermoluminescence studies of Ba_2_ZnSi_2_O_7_:1 mol% Tb^3+^, *z* Dy^3+^ (*z* = 1, 1.25, 1.5, 1.75, 2 mol%) phosphors

The TL glow curves of Ba_2_ZnSi_2_O_7_: 1 mol% Tb^3+^, zDy^3+^ (*z* = 1, 1.25, 1.5, 1.75, and 2 mol%) samples exposed to a γ-dose of 1 KGy are shown in Fig. 11. Two distinct glow peaks at around 395 K and 497 K are shown by the observations, which were performed at a heating rate of 2.85 K s^−1^. Due to the increased availability of luminous centers and enhanced energy transfer from traps, it is clear that the TL intensity rises with increasing Dy^3+^ concentration up to 1.75 mol%.^44^ The basic defect-assisted trapping process covered in Section 3.1, where co-doping alters the concentration and distribution of lattice trapping centers, can account for the fluctuation in TL intensity and trap characteristics with Tb^3+^/Dy^3+^ concentration.

**Fig. 11:**
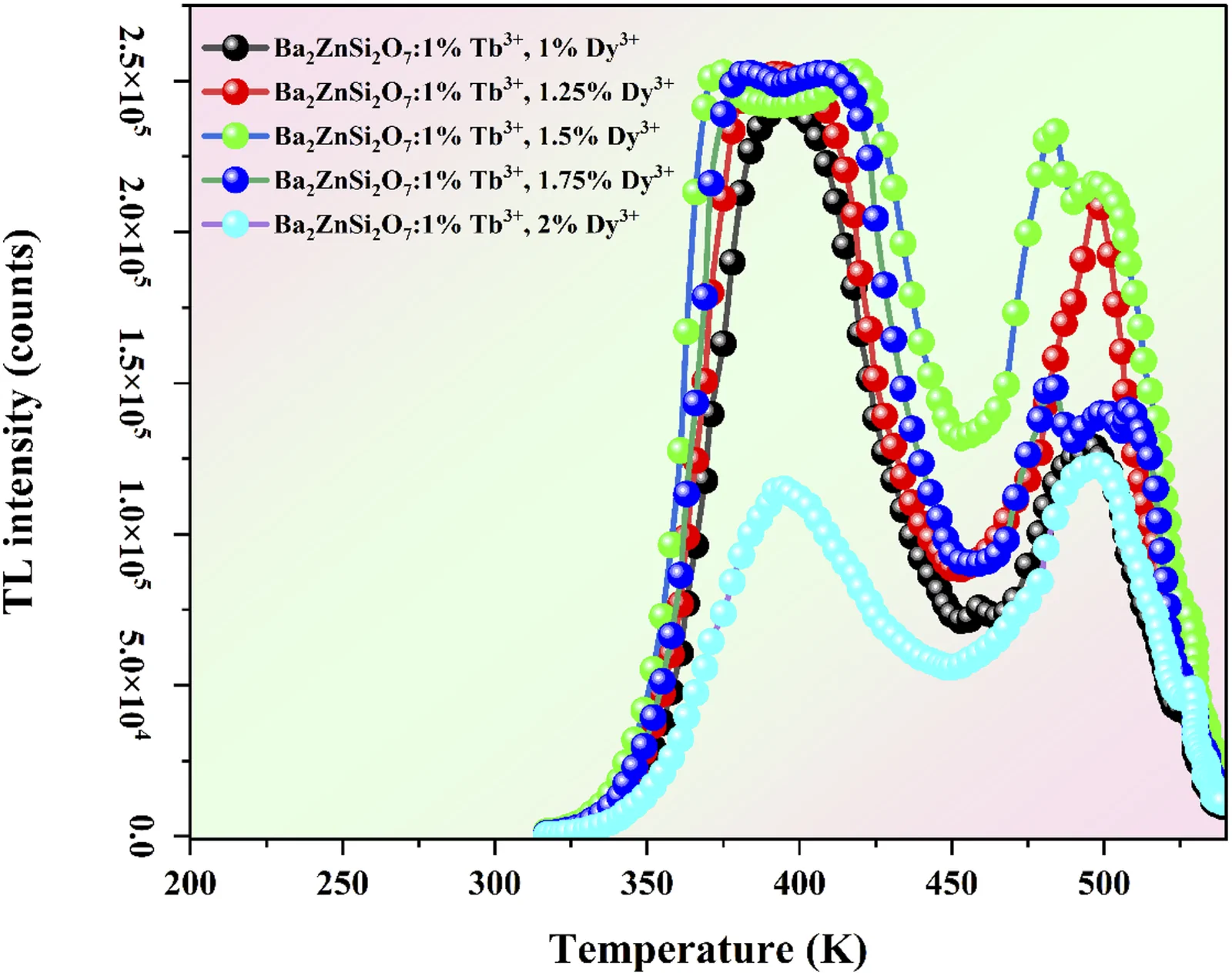
TL glow curve at 1 KGy dose for different concentrations of Dy^3+^ in Ba_2_ZnSi_2_O_7_: 1 mol% Tb^3+^.

Fig. 12 illustrates the TL glow curves of the optimized composition, Ba_2_ZnSi_2_O_7_:1 mol% Tb^3+^,1.75 mol% Dy^3+^ exposed to γ-radiation doses ranging from 25 Gy to 10 KGy. A well-defined peak around 497 K is observed at lower dosage and higher doses peak shifted to lower temperature with the same heating rate of 2.85 K s^−1^. After 7000 Gy the glow curve saturates. As shown in Fig. 13, the TL intensity increases linearly with dose in the range of 1–7KGy. Beyond 7 KGy, the glow curve begins to show saturation behaviour, indicating that 7000 Gy can be considered an optimal dose for further studies.

**Fig. 12:**
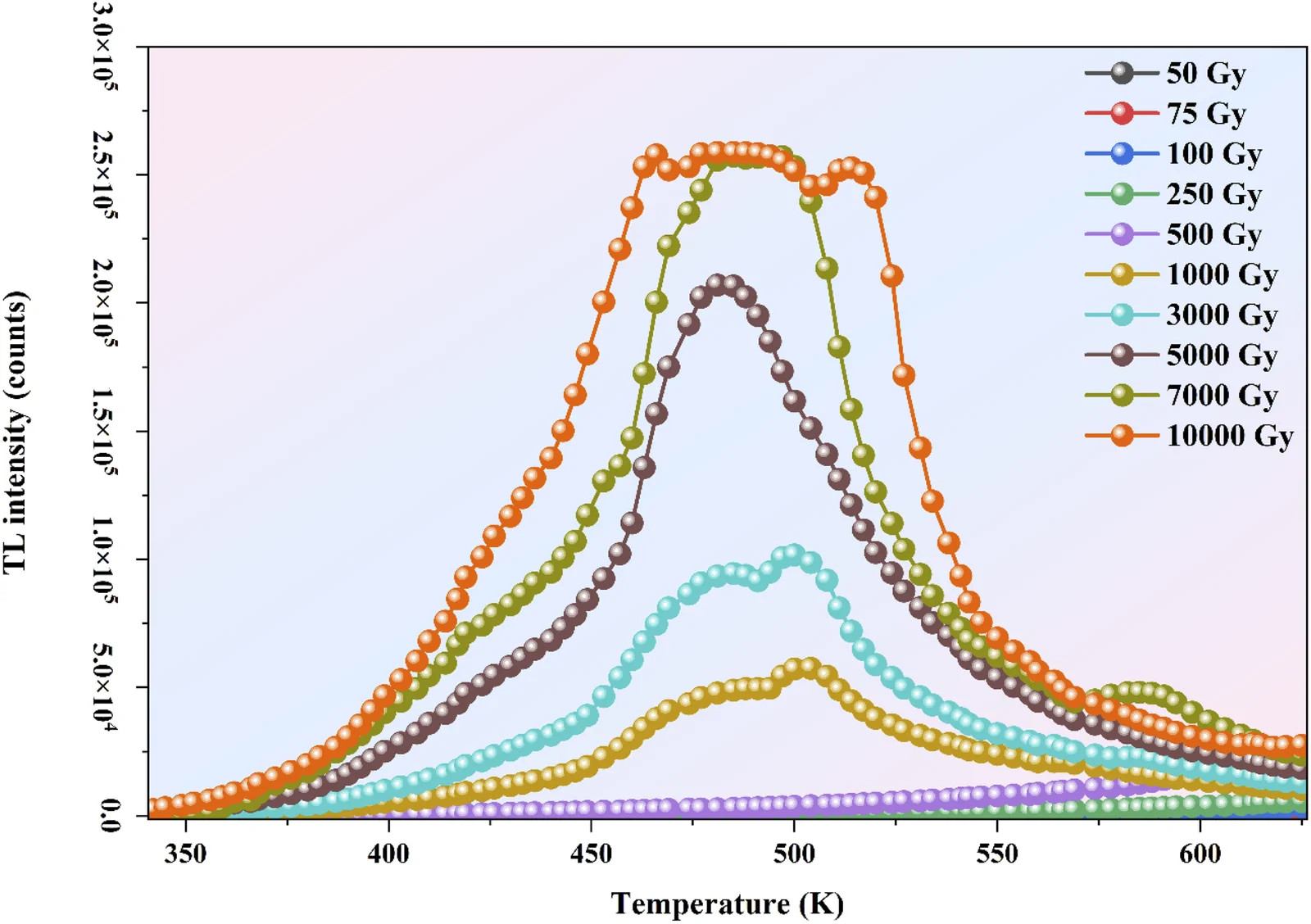
TL glow curve of Ba_2_ZnSi_2_O_7_:1 mol% Tb^3+^,1.75 mol% Dy^3+^ at different doses.

**Fig. 13:**
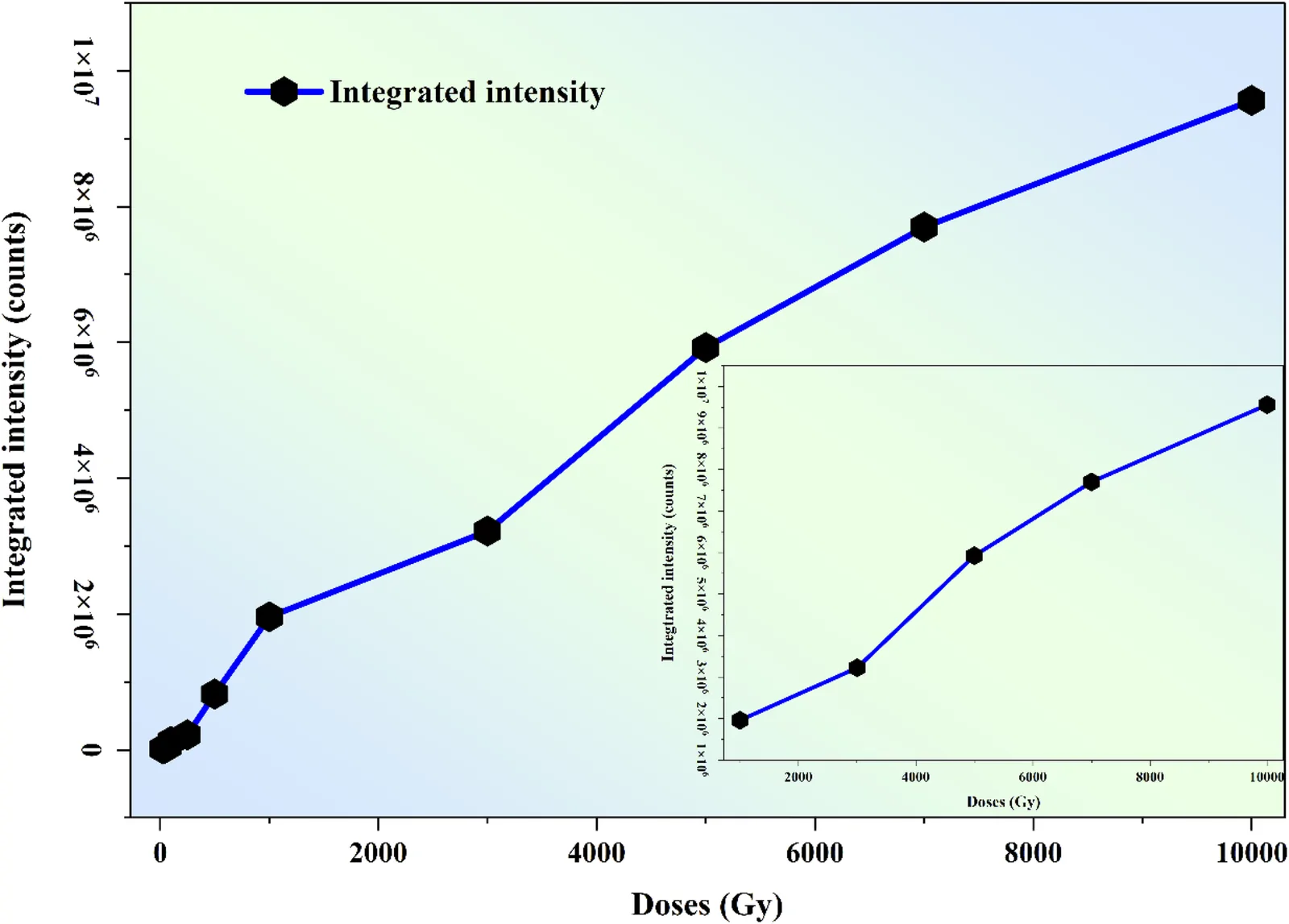
TL intensity variation with different doses.

The variation of total TL intensity with γ-dose for Ba_2_ZnSi_2_O_7_:1 mol% Tb^3+^,1.75 mol% Dy^3+^ is also depicted in Fig. 14. The increase in TL intensity with dose is attributed to the higher population of charge carriers trapped within defect sites created by irradiation. The inset of Fig. 14 clearly highlights a linear response region between 1 KGy and 7 KGy, as well as at doses beyond 7 KGy saturation effects.^45^ The integrated TL intensity represents the total emitted light, obtained by calculating the area under the glow curve over the entire temperature range.

**Fig. 14:**
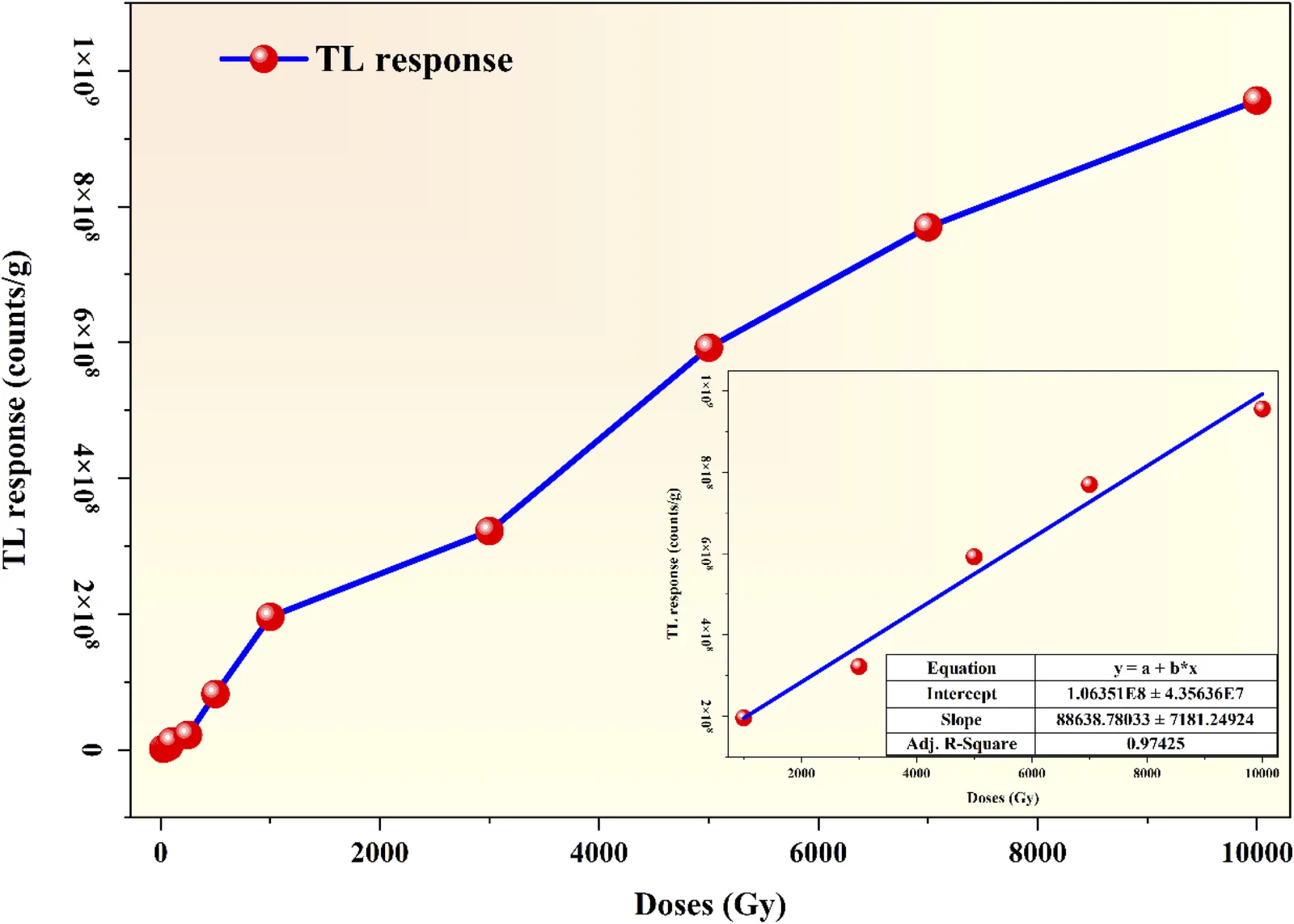
TL response plot for Ba_2_ZnSi_2_O_7_:1 mol% Tb^3+^, 1.75 mol% Dy^3+^ phosphor at different doses.

#### 3.3.1. Sensitivity

This parameter depends on factors such as the type of radiation, the experimental setup, and the characteristics of the dosimetric material. For Ba_2_ZnSi_2_O_7_:1 mol% Tb^3+^,1.75 mol% Dy^3+^, the sensitivity was determined from the linear region 1 KGy – 7 KGy of the dose–response curve shown in Fig. 14. The calculated sensitivity is 88 638.78 counts g^−1^ Gy^−1^.

#### 3.3.2. Minimum detectable dose

MDD, defined eqn (1). For the Ba_2_ZnSi_2_O_7_:1 mol% Tb^3+^,1.75 mol% Dy^3+^ phosphor, the MDD was found to be 31.374 mGy, indicating its capability to detect relatively low levels of radiation.

#### 3.3.3. Kinetic parameters

##### 3.3.3.1. CGCD method

The glow curve analysis of Ba_2_ZnSi_2_O_7_:1 mol% Tb^3+^,1.75 mol% Dy^3+^ phosphor under gamma irradiation suggests a complex recombination mechanism, where charge carriers may undergo re-trapping before finally recombining. To extract meaningful information about the trapping states, the glow curves were analysed using the Computerized Glow Curve Deconvolution (CGCD) method.^46^Fig. 15 displays the fitted glow curves for the optimal Dy^3+^ concentration at various irradiation doses. Table 3 lists the calculated kinetic parameters, such as the *s*, *E*, *τ*, and *b*. TL emission is caused by rather deep trapping centers, as shown by the activation energy values, which vary from 1 to 1.79 eV.

**Fig. 15:**
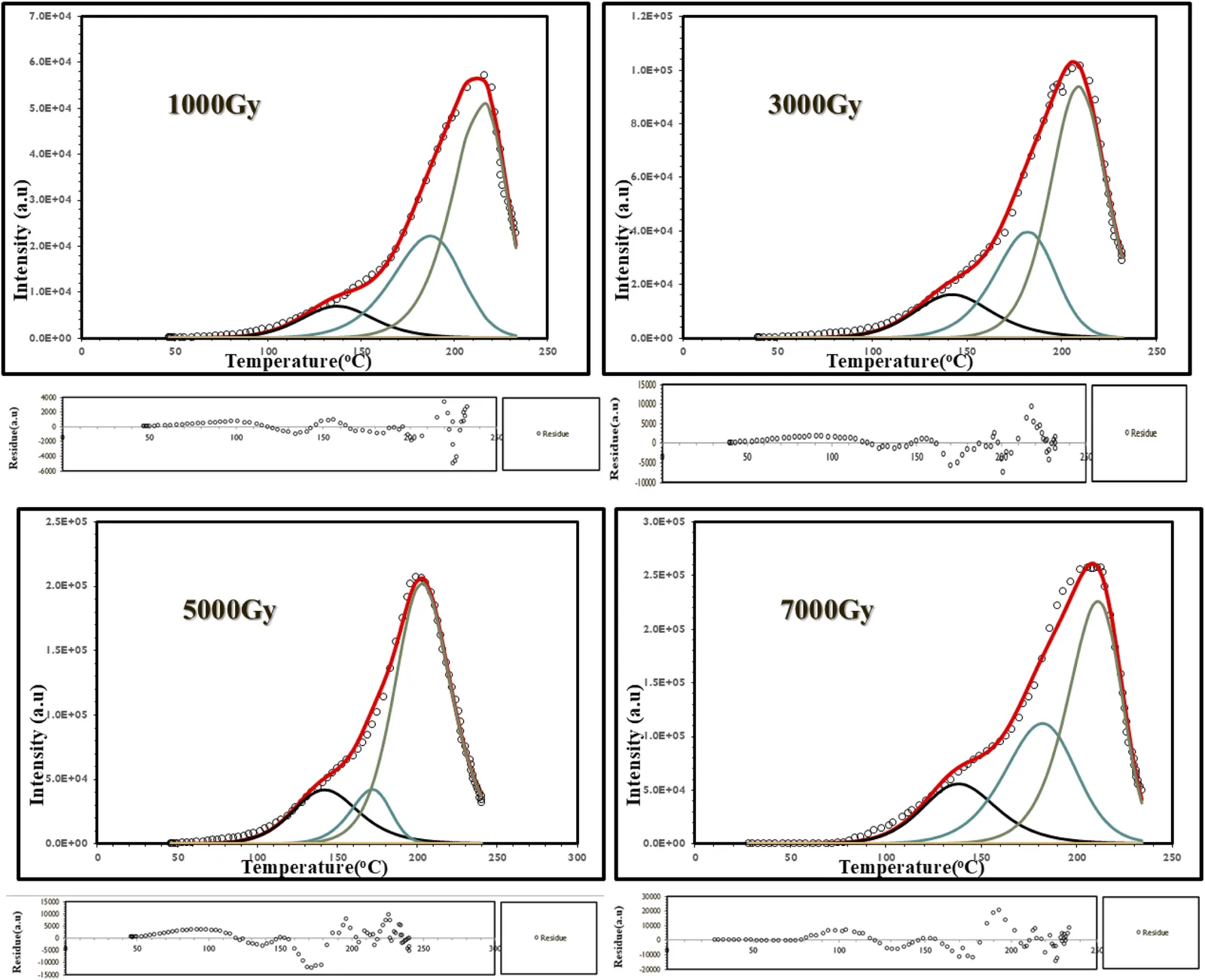
Fitted TL glow curve peaks of Ba_2_ZnSi_2_O_7_:1 mol% Tb^3+^, 1.75 mol% Dy^3+^ phosphor.

**Table 3:** Trap energy values of Ba_2_ZnSi_2_O_7_:1 mol% Tb^3+^, 1.75 mol% Dy^3+^ phosphor evaluated using different analytical methods

Doses (Gy)	Peak number	*T*_m_ (K)	CGCD					Chen's		Initial rise
			*E* (eV)	*b*	FF (s^−1^)	Lifetime (days)	FOM (%)	*E* (eV)	FF (s^−1^)	*E* (eV)
1000	1	410.03	1.00	1.85	3.64 × 10^11^	5.05	4.99	1.00	3.31 × 10^11^	0.98
	2	460.68	1.10	1.27	1.90 × 10^11^	508.31		1.07	7.66 × 10^10^	1.09
	3	489.31	1.53	1.22	1.33 × 10^15^	1 809 601.21		1.35	1.41 × 10^13^	1.52
3000	1	415.49	1.01	1.99	3.34 × 10^11^	8.18	4.97	0.98	1.22 × 10^11^	0.99
	2	455.53	1.27	1.32	2.32 × 10^13^	3496.35		1.17	1.49 × 10^12^	1.26
	3	482.98	1.79	1.76	1.28 × 10^18^	55 789 905.69		1.50	9.75 × 10^14^	1.78
5000	1	415.69	1.01	1.99	3.34 × 10^11^	8.18	4.67	0.96	6.90 × 10^10^	0.99
	2	445.43	1.55	1.30	9.14 × 10^16^	58 145.18		1.47	1.09 × 10^16^	1.54
	3	476.84	1.57	1.99	9.14 × 10^15^	1 283 923.82		1.48	9.41 × 10^14^	1.56
7000	1	411.43	1.05	1.99	1.45 × 10^12^	9.19	4.85	1.00	3.64 × 10^11^	1.04
	2	455.38	1.14	1.45	7.49 × 10^11^	628.71		1.13	4.97 × 10^11^	1.13
	3	484.43	1.56	1.20	3.79 × 10^15^	2 083 691.93		1.51	1.12 × 10^15^	1.55

##### 3.3.3.2. Chen's peak shape method

To further validate these findings, Chen's peak shape method was applied using the formalism developed by Kitis. This method relies on three characteristic temperatures *T*_1_, *T*_m_, and *T*_2_ obtained from the glow curve. The relevant plots are illustrated in Fig. S9–S12. From these parameters, the symmetry factor (*µ*_g_) was evaluated and found to lie between 0.41 and 0.51.^47^ This range clearly indicates that the TL process predominantly follows first-order kinetics. The trapping parameters were then calculated using Kitis' expressions, and the average activation energy values were found to closely match those obtained from the CGCD analysis, as presented in Table 3.

##### 3.3.3.3. Initial rise method

This approach considers only the low-temperature portion of the glow curve, where the intensity follows an exponential trend. By plotting ln(*I*) against 1/*kT*, a straight line is obtained, and the activation energy is derived from its slope. The corresponding linear fits for the initial rise region are shown in Fig. S9–S12. The activation energies calculated using this method is listed in Table 3. The activation energies are well matching with all other methods to calculate the activation energy also.

### 3.4. Fading analysis

To check the applicability of the optimized co-doped Ba_2_ZnSi_2_O_7_ phosphors for long-term radiations dosimetry, their fading behaviour of thermoluminescence was investigated for the storage time of 90 days at room temperature. The normalized TL intensity (%) *vs.* storage time is shown in Fig. 16, in which all three phosphors show a similar initial decrease in TL intensity over the first few days, followed by a comparatively slower decay that is characteristic of the release of charge carriers from shallow trapping centers. Based on the investigated compositions, the best fading stability about 70% of initial TL intensity after 90 days is observed for Ba_2_ZnSi_2_O_7_:0.4 mol% Sm^3+^, 1.25 mol% Tb^3+^. This means that there are relatively deep and stable trapping centres which efficiently suppress the thermal de-trapping of charge carriers during storage. The Ba_2_ZnSi_2_O_7_:1.0 mol% Tb^3+^, 1.75 mol% Dy^3+^ phosphor has moderate fading with the retention rate of ∼48–50% after 90 days. In contrast, the fading of Ba_2_ZnSi_2_O_7_:1.5 mol% Dy^3+^, 0.5 mol% Sm^3+^ is larger, and the TL intensity drops to almost 15% after 90 days, indicating that there is a higher proportion of shallow traps and lower charge storage stability. This fading effect is caused by variations in the distribution of traps and in the depths of the traps caused by the introduction of rare earth co-dopants. In deep traps, charge is more difficult to release and it is more stable, while in shallow traps, ambient temperature releases the trapped carriers. Thus, the phosphor of Sm^3+^/Tb^3+^ co-doped is the best suited for the long-term TL dosimetry applications, due to its high storage stability.

**Fig. 16:**
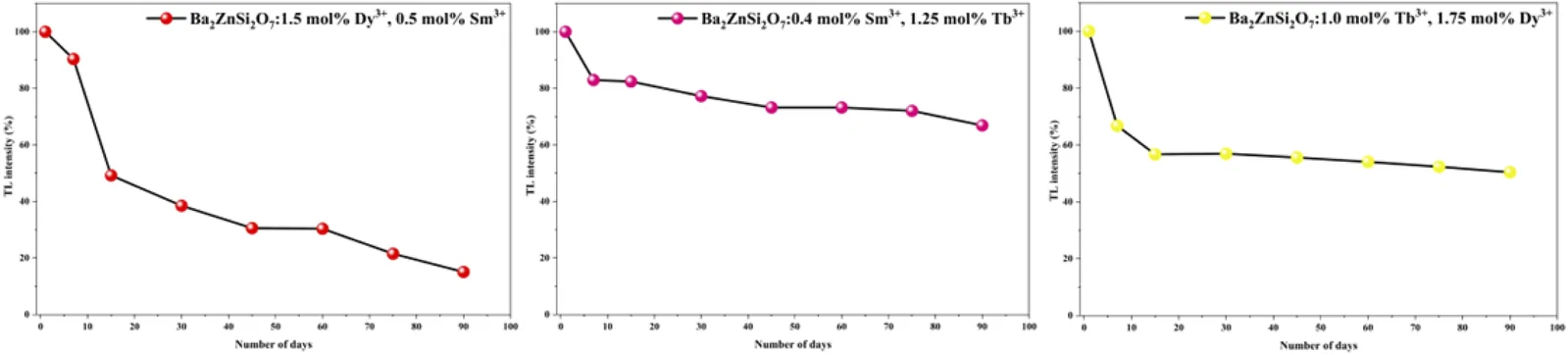
Fading analysis of the (a) Dy^3+^/Sm^3+^ (b) Sm^3+^/Tb^3+^ (c) Tb^3+^/Dy^3+^ in Ba_2_ZnSi_2_O_7_ phosphors.

The thermoluminescence properties of Ba_2_ZnSi_2_O_7_ phosphors co-doped with various rare-earth ions show a clear dependence on the type and concentration of dopants, as well as the applied irradiation dose. The observed TL response can be explained through the formation of trapping centers, the movement of charge carriers, and the energy transfer processes occurring within the host lattice. Analysis of the glow curves reveals distinct and well-defined peaks, which indicate the presence of specific trapping levels in the material. Detailed deconvolution and parameter evaluation show that the activation energies mainly fall within the range of approximately 1–1.8 eV. These values are characteristic of deep traps, which play an important role in improving thermal stability and minimizing signal fading over time. Such features are highly desirable for dosimetric applications, especially when long-term storage of the TL signal is required.^48–50^ Moreover, the close agreement in activation energy values obtained from different methods such as CGCD, Chen's peak shape approach, and the initial rise technique adds confidence to the accuracy of the trapping analysis. Dopant ions significantly influence the TL performance of the material. Rare-earth ions like Tb^3+^, Dy^3+^, and Sm^3+^ not only act as luminescent centers but also modify the trapping structure.^47,51^ At optimal doping levels, they enhance TL intensity by increasing the number of recombination centers and facilitating efficient energy transfer from traps. However, when the dopant concentration exceeds a certain limit, concentration quenching occurs. This is mainly due to the reduced distance between neighbouring ions, which promotes non-radiative interactions and leads to a decline in TL intensity. This emphasizes the need for careful optimization of dopant concentration to achieve the best performance. Among the investigated compositions, Ba_2_ZnSi_2_O_7_:1.5 mol% Dy^3+^, 0.5 mol% Sm^3+^ exhibits strong TL emission at higher doses, making it well-suited for high-dose applications. On the other hand, Ba_2_ZnSi_2_O_7_:0.4 mol% Sm^3+^, 1.25 mol% Tb^3+^ demonstrates higher sensitivity and a lower minimum detectable dose, suggesting its usefulness in moderate-dose monitoring. Additionally, Ba_2_ZnSi_2_O_7_:1 mol% Tb^3+^, 1.75 mol% Dy^3+^ shows a wide linear response range along with good sensitivity, making it a promising candidate for intermediate dose measurements. In summary, the TL behaviour of these phosphors is governed by a combination of deep and moderately deep trapping centers, along with efficient luminescence facilitated by rare-earth doping. The strong agreement between different analytical techniques, along with stable trapping characteristics and adjustable dose–response behaviour, demonstrates that Ba_2_ZnSi_2_O_7_ based phosphors are highly promising materials for a wide range of radiation dosimetry applications.

## 4. Conclusion

In this work, the thermoluminescence behaviour of Ba_2_ZnSi_2_O_7_ phosphors co-doped with different rare-earth ions was systematically studied to assess their potential for radiation dosimetry. The TL glow curves exhibited well-defined peaks, indicating the presence of distinct trapping centers within the material. The trapping parameters evaluated using CGCD, Chen's peak shape method, and the initial rise method showed good agreement, confirming the reliability of the analysis. The calculated activation energies are ∼1–1.8 eV suggest the existence of deep traps, which are essential for ensuring good thermal stability and minimal signal fading. The TL intensity was found to increase with increasing gamma dose, showing a linear response over specific dose ranges, followed by saturation at higher doses due to trap filling. The results also highlight the strong influence of dopant type and concentration on TL performance, with optimal doping leading to enhanced intensity, while higher concentrations resulted in quenching effects. Among the studied samples, different compositions exhibited suitability for varying dose ranges, including high-dose, intermediate, and moderate-dose applications. Overall, these findings demonstrate that Ba_2_ZnSi_2_O_7_ based phosphors are promising materials for TL dosimetry, with tuneable properties that can be optimized for diverse radiation detection applications.

## Author contributions

Tejas: methodology, formal analysis, validation, investigation, data curation, writing – original draft, writing – review & editing. Naregundi Karunakara: visualization, software, resources. Nagaraj Kamath: validation, investigation. Sudha D. Kamath: writing – review & editing, visualization, validation, supervision, resources, project administration, investigation, formal analysis, data curation.

## Conflicts of interest

The authors declare that they have no known competing financial interests or personal relationships that could have appeared to influence the work reported in this paper.

## Supplementary Material

RA-016-D6RA07885K-s001

## Data Availability

The data that support the findings of this study are available from the corresponding author upon reasonable request. Supplementary information (SI) is available. See DOI: https://doi.org/10.1039/d6ra07885k.
