# Supplementary material for: Engineering trap dynamics in Ba2ZnSi2O7 phosphors: a study on rare-earth co-doping for efficient TL dosimetry
Source: RSC Adv. 2026 Sep 14;16(44):54751–68. doi: 10.1039/d6ra07885k (PMC13572922; doi:10.1039/d6ra07885k)
Supplement: Supplementary file 1 [file RA-016-D6RA07885K-s001.pdf]

Supplementary Information (SI) for RSC Advances.
This journal is © The Royal Society of Chemistry 2026

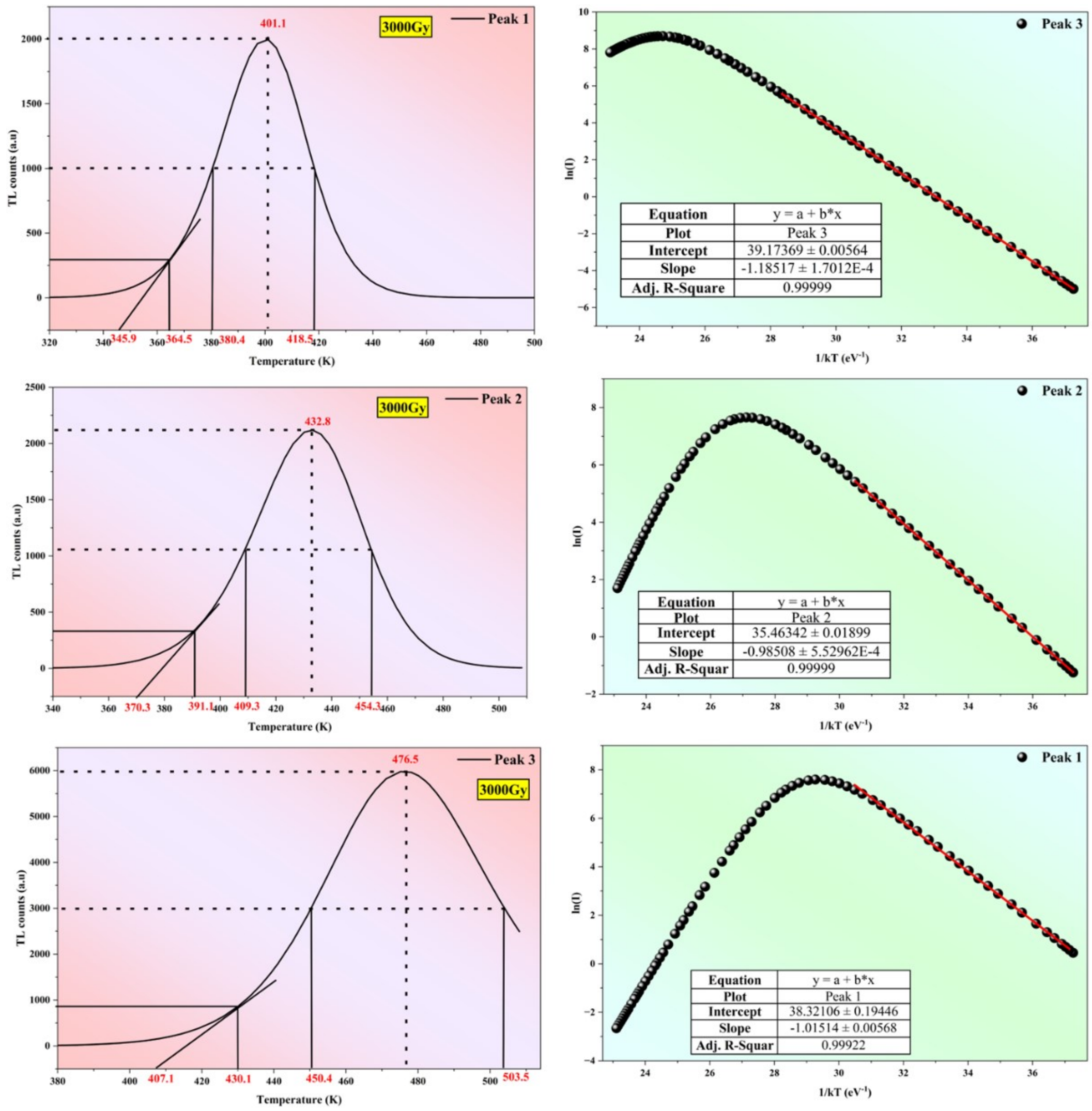

Figure S1. TL glow curve analysis of deconvoluted peaks of $Ba_2ZnSi_2O_7$:1.5 mol % Dy^{3+}, 0.5 mol% Sm^{3+} phosphor for 3000 Gy with ln(I) v/s 1/kT plot respectively.

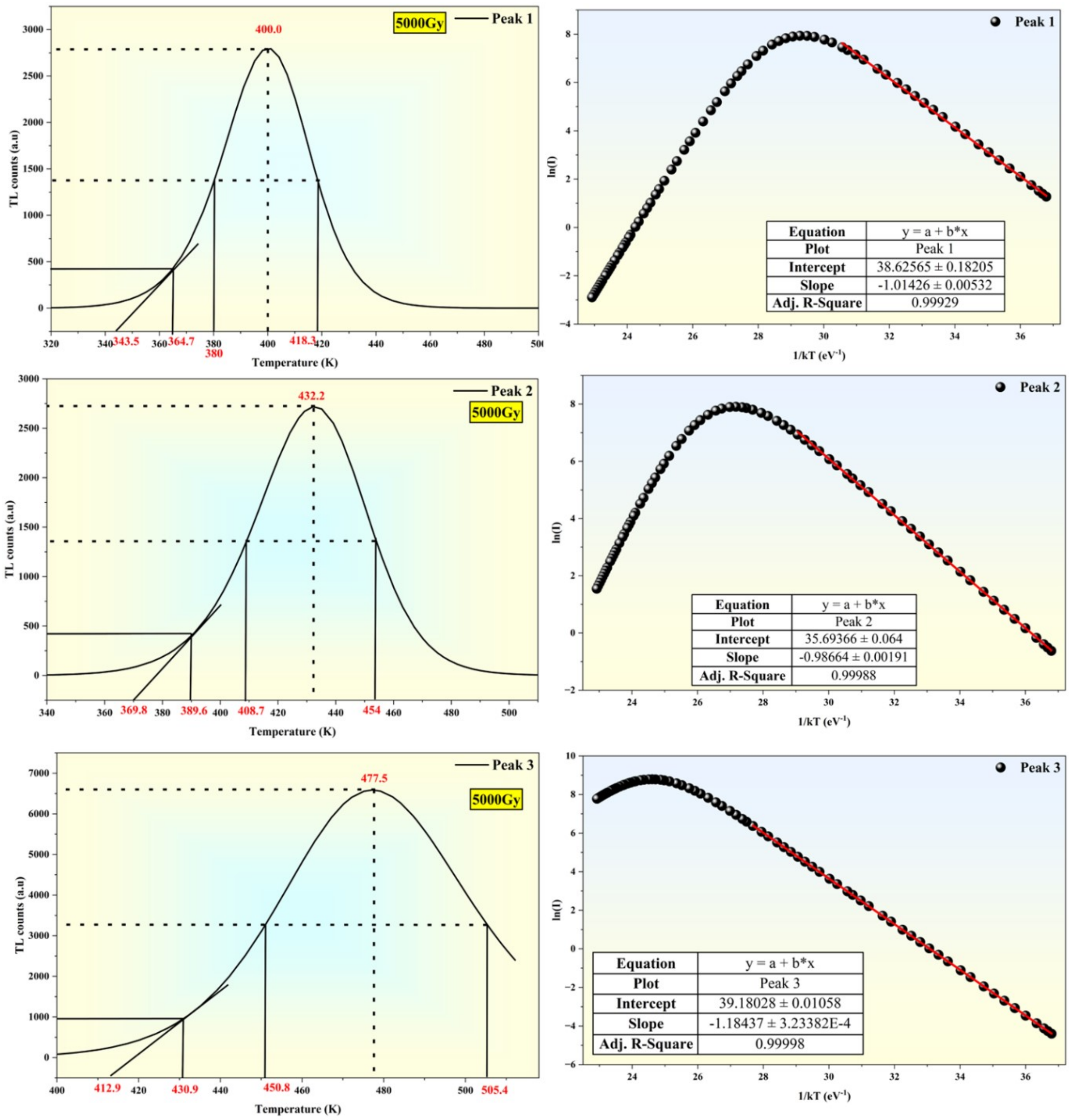

Figure S2. For the $Ba_2ZnSi_2O_7$ phosphor containing 1.5 mol% Dy^{3+} and 0.5 mol% Sm^{3+}, the glow curve at 5 kGy and the corresponding ln(I) against 1/kT plot.

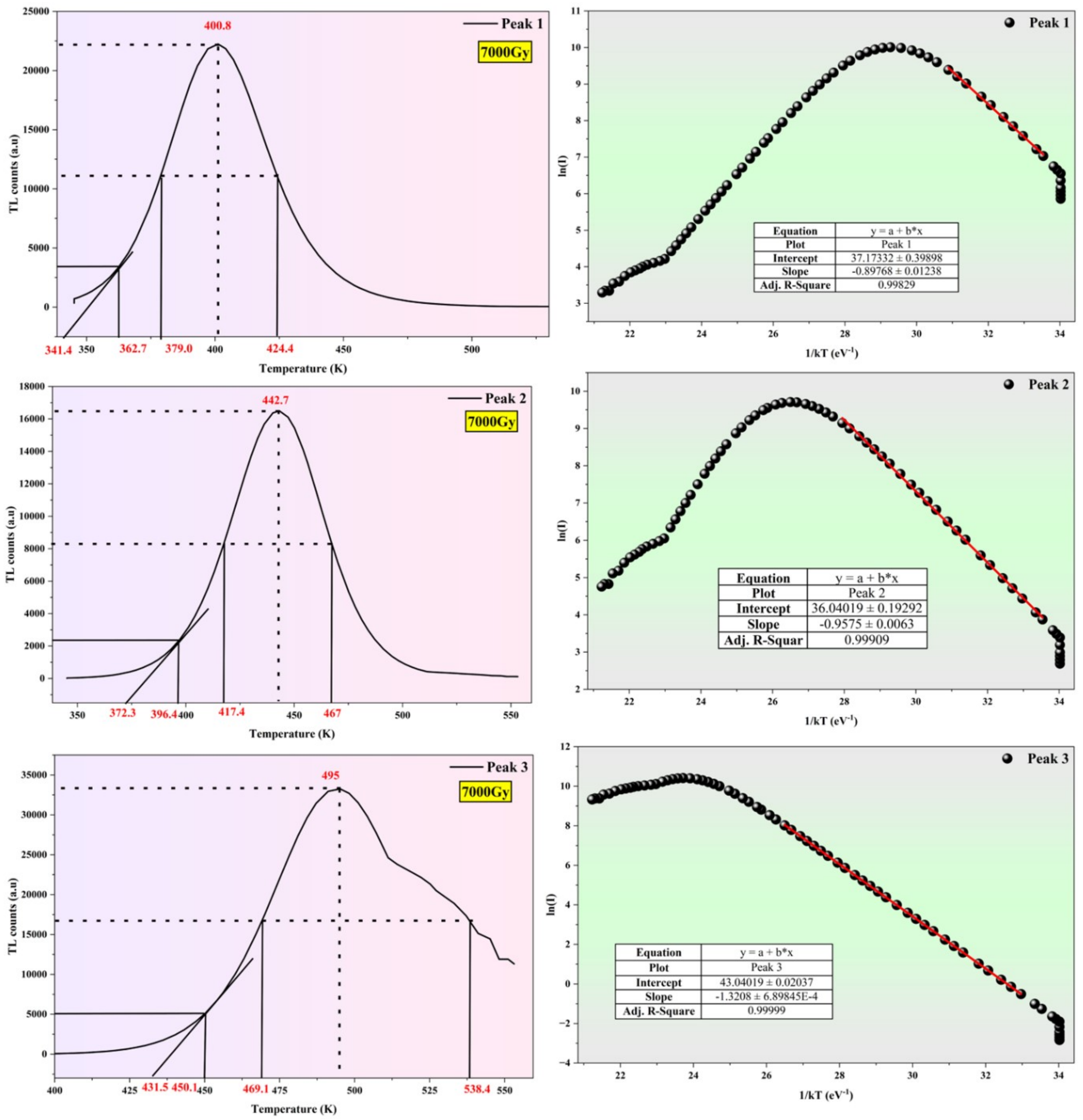

Figure S3. The deconvoluted TL glow peaks of $Ba_2ZnSi_2O_7$:1.5 mol% Dy^{3+}, 0.5 mol% Sm^{3+} at 7000 Gy along with the ln(I) vs 1/kT plot to understand the trapping parameters.

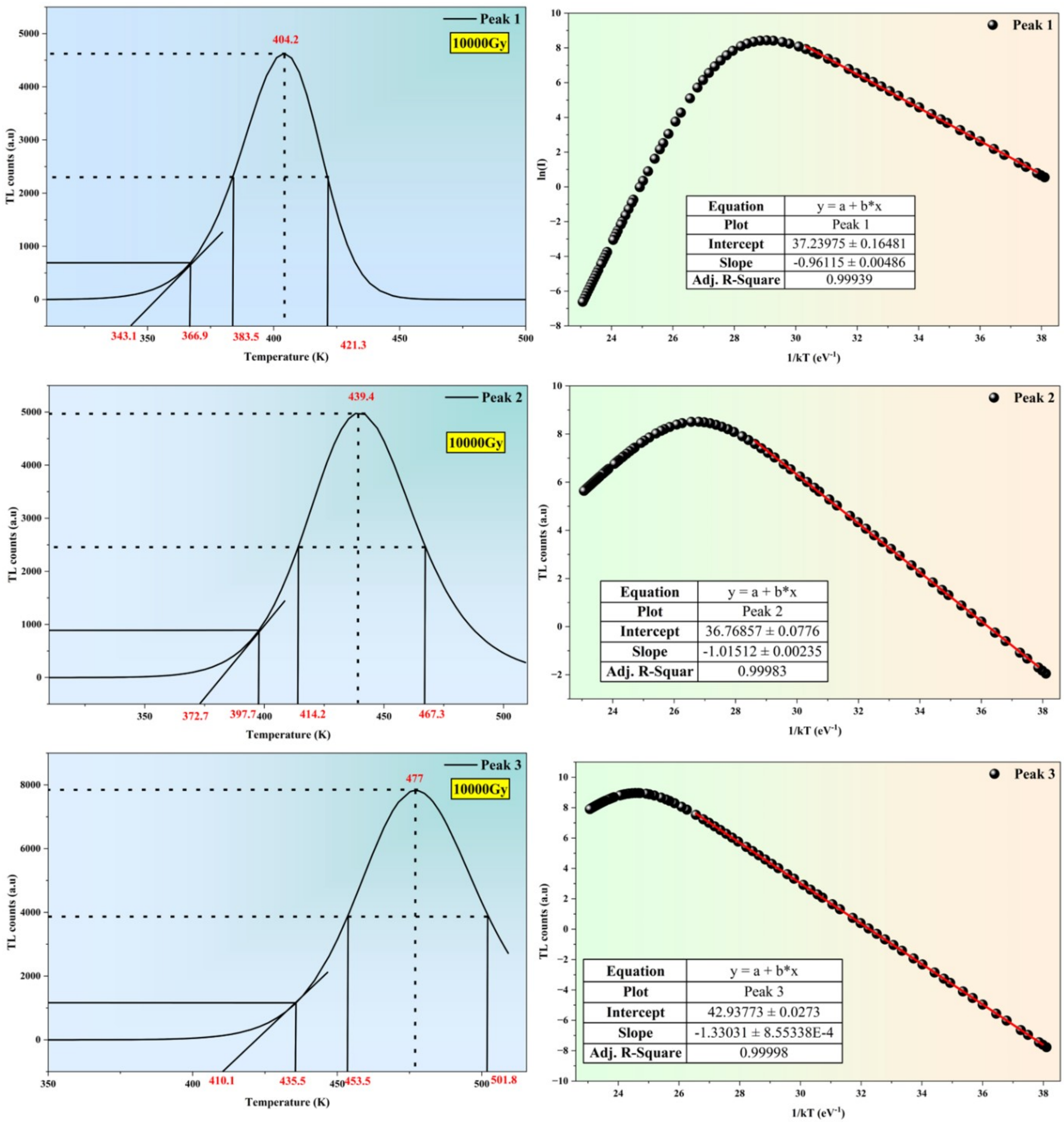

Figure S4. Deconvolution analysis of the TL glow curve for $Ba_2ZnSi_2O_7$:1.5 mol% Dy^{3+}, 0.5 mol% Sm^{3+} phosphor irradiated at 10 kGy was carried out, and the related ln(I) vs 1/kT graph was examined.

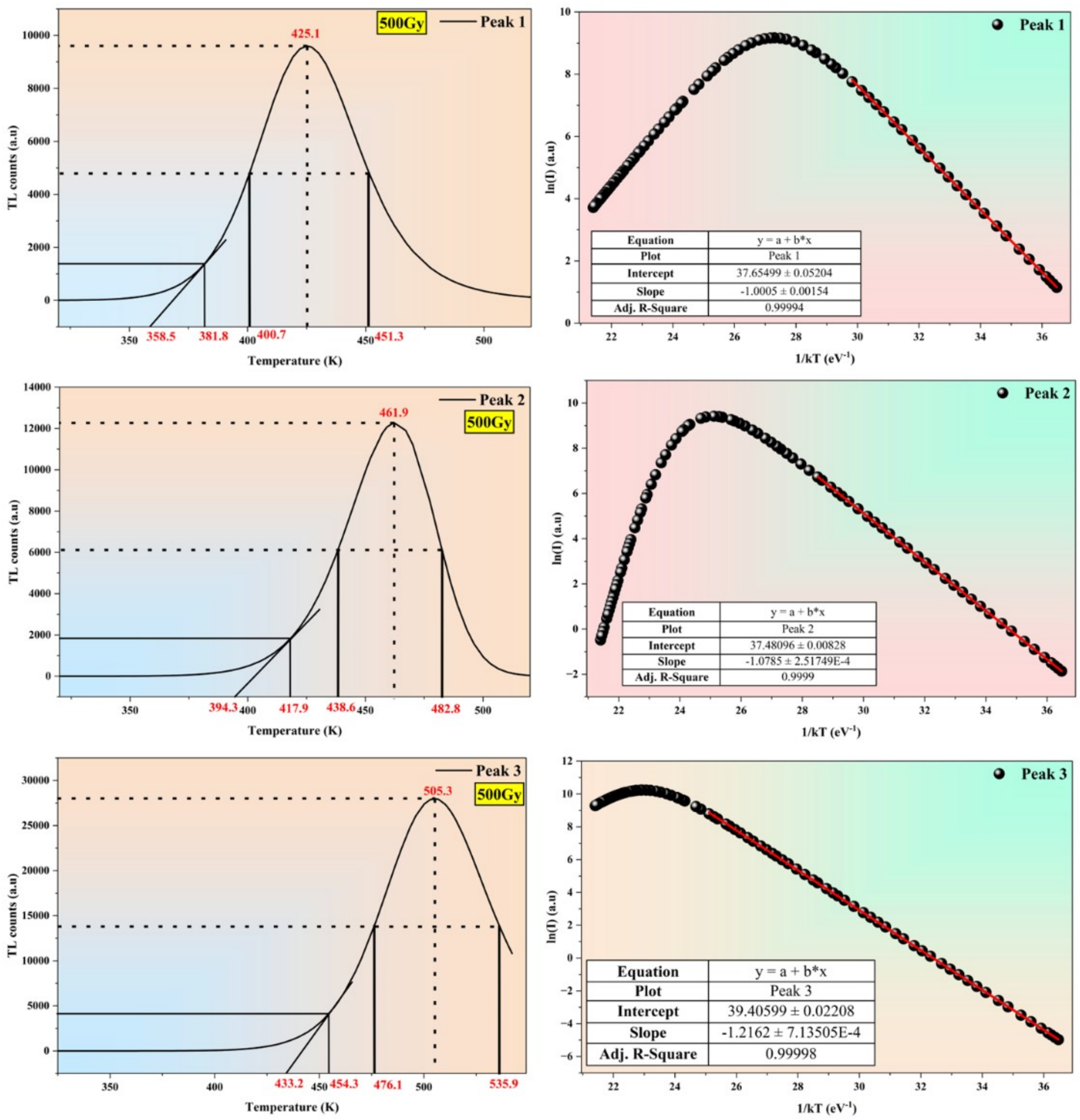

Figure S5. Analysis of TL glow curves for the deconvoluted peaks of $Ba_2ZnSi_2O_7$:0.4 mol% Sm^{3+}, 1.25 mol% Tb^{3+} phosphor at 500 Gy, showing the corresponding ln(I) versus 1/kT plot.

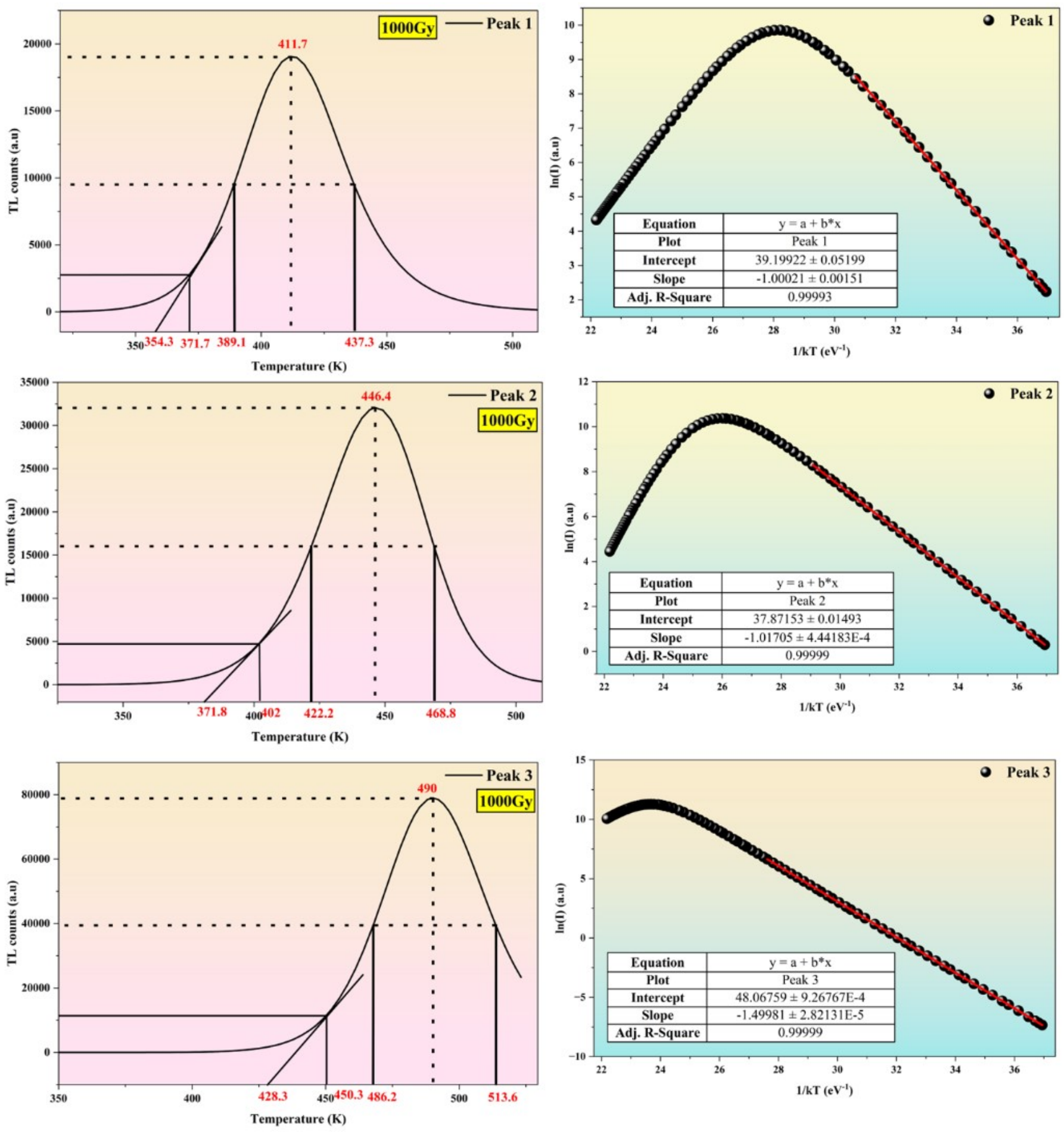

Figure S6. Deconvolution analysis of the TL glow curve for $Ba_2ZnSi_2O_7$: 0.4 mol% Sm^{3+}, 1.25 mol% Tb^{3+} phosphor irradiated at 1000 Gy, along with the corresponding ln(I) versus 1/kT plot.

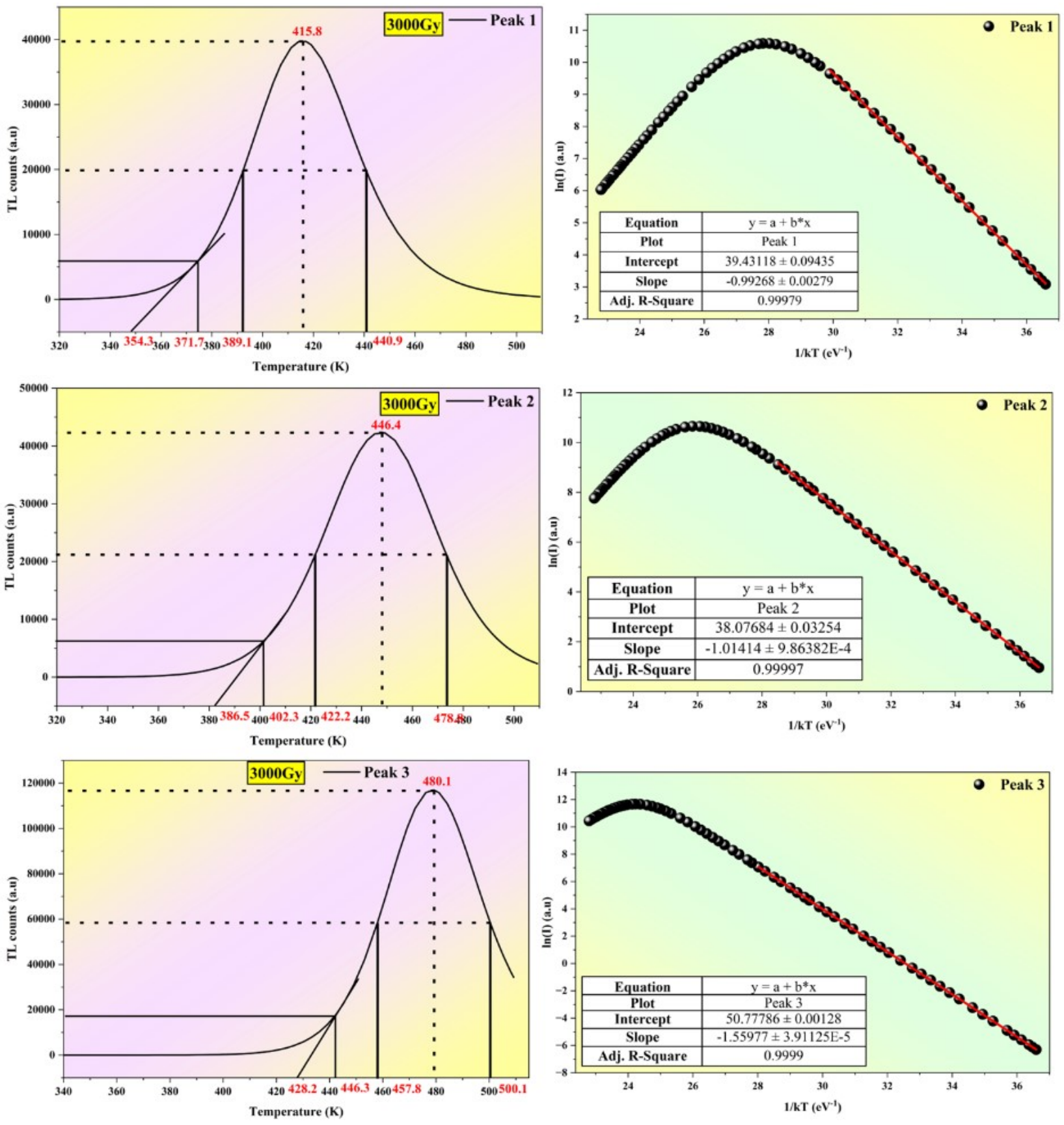

Figure S7. TL glow curve of $Ba_2ZnSi_2O_7$ doped with 0.4 mol% Sm^{3+}, 1.25 mol% Tb^{3+} at 3000 Gy, showing deconvoluted peaks and the associated ln(I) vs 1/kT representation.

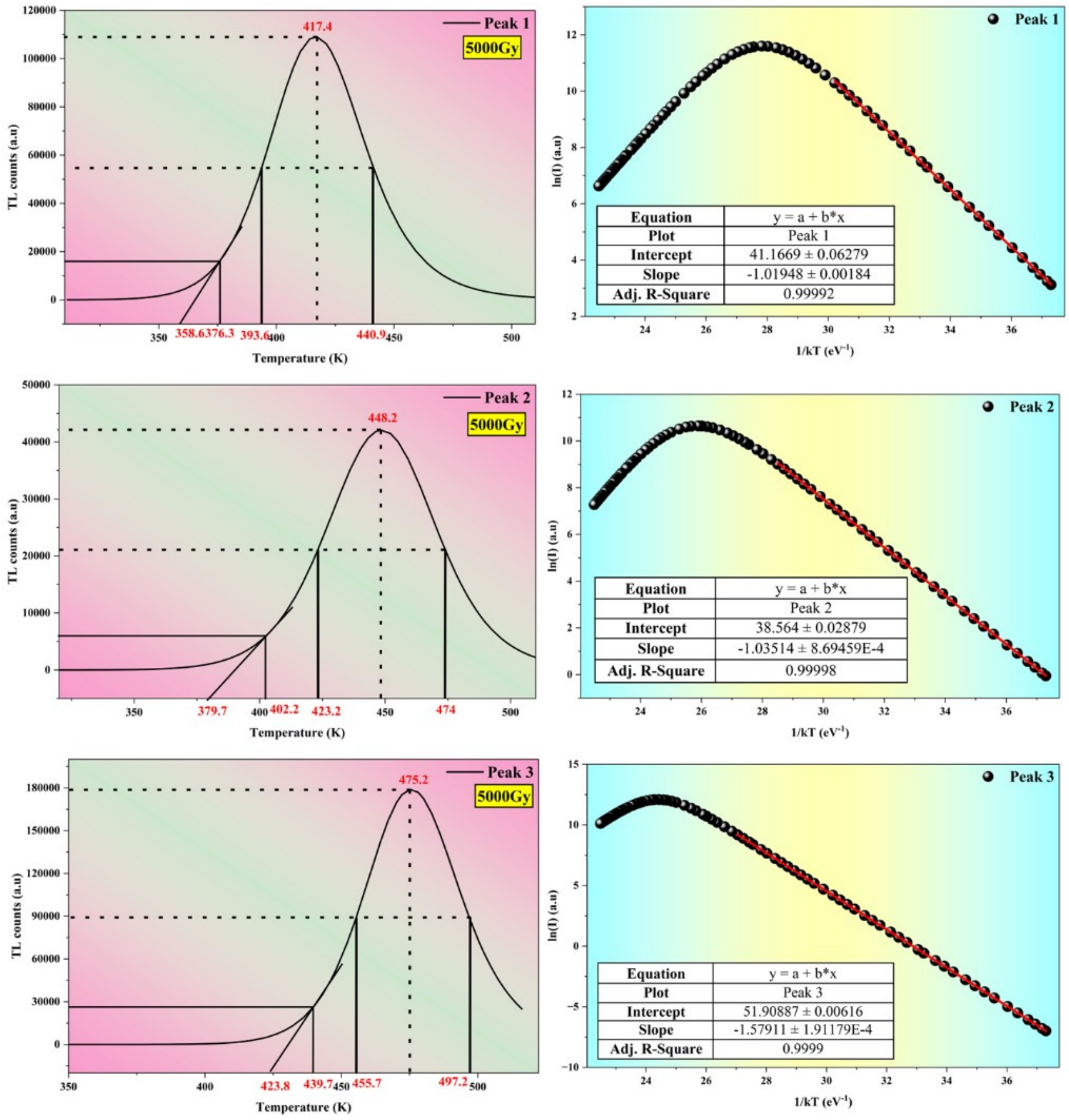

Figure S8. Deconvoluted thermoluminescence glow peaks of $Ba_2ZnSi_2O_7$: 0.4 mol% Sm^{3+}, 1.25 mol% Tb^{3+} phosphor at a dose of 5000 Gy, with ln(I) versus 1/kT plot.

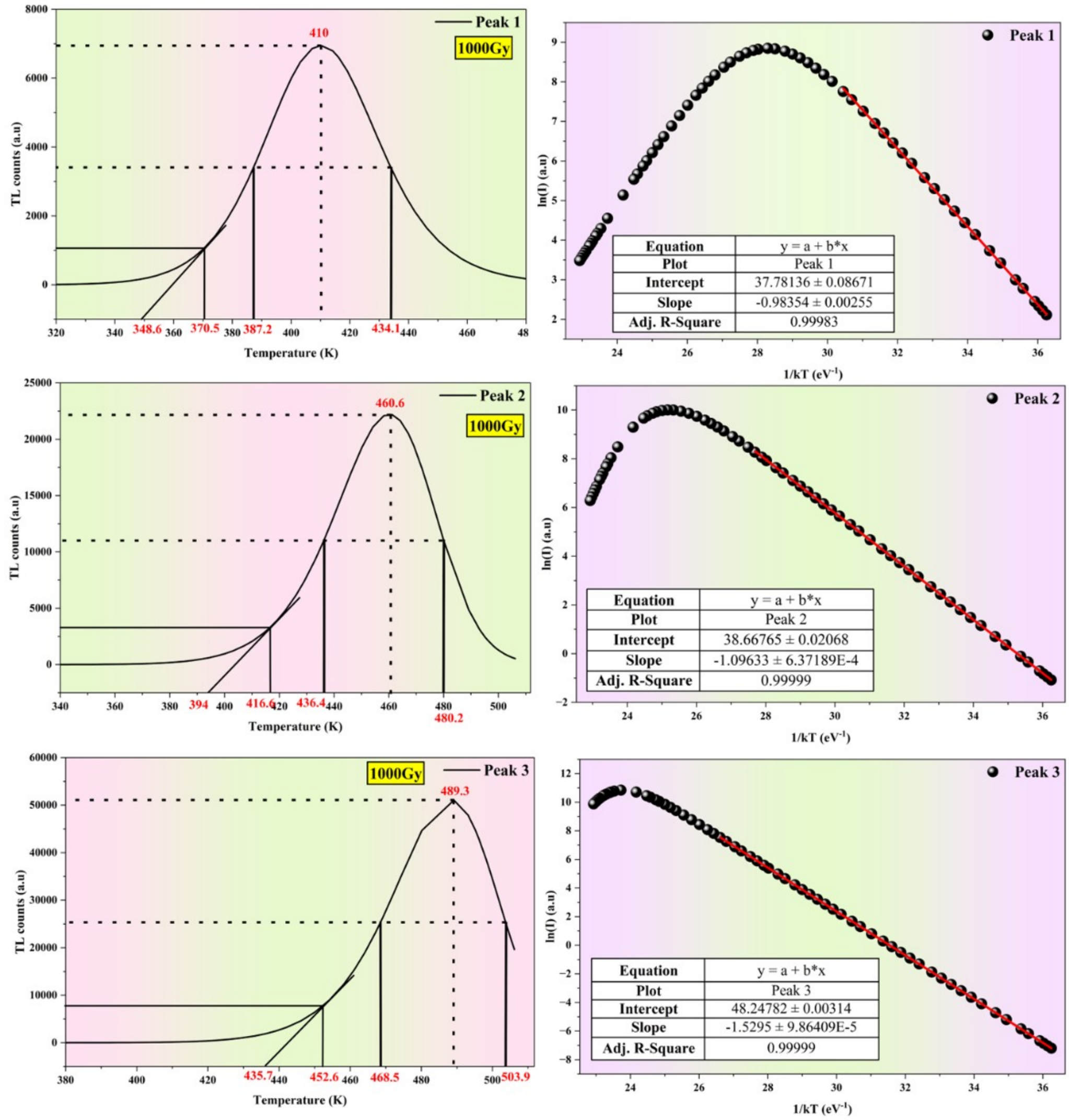

Figure S9. Deconvoluted TL glow curve of $Ba_2ZnSi_2O_7$:1 mol% Tb^{3+},1.75 mol% Dy^{3+} phosphor at 1000 Gy and ln(I) versus 1/kT plot.

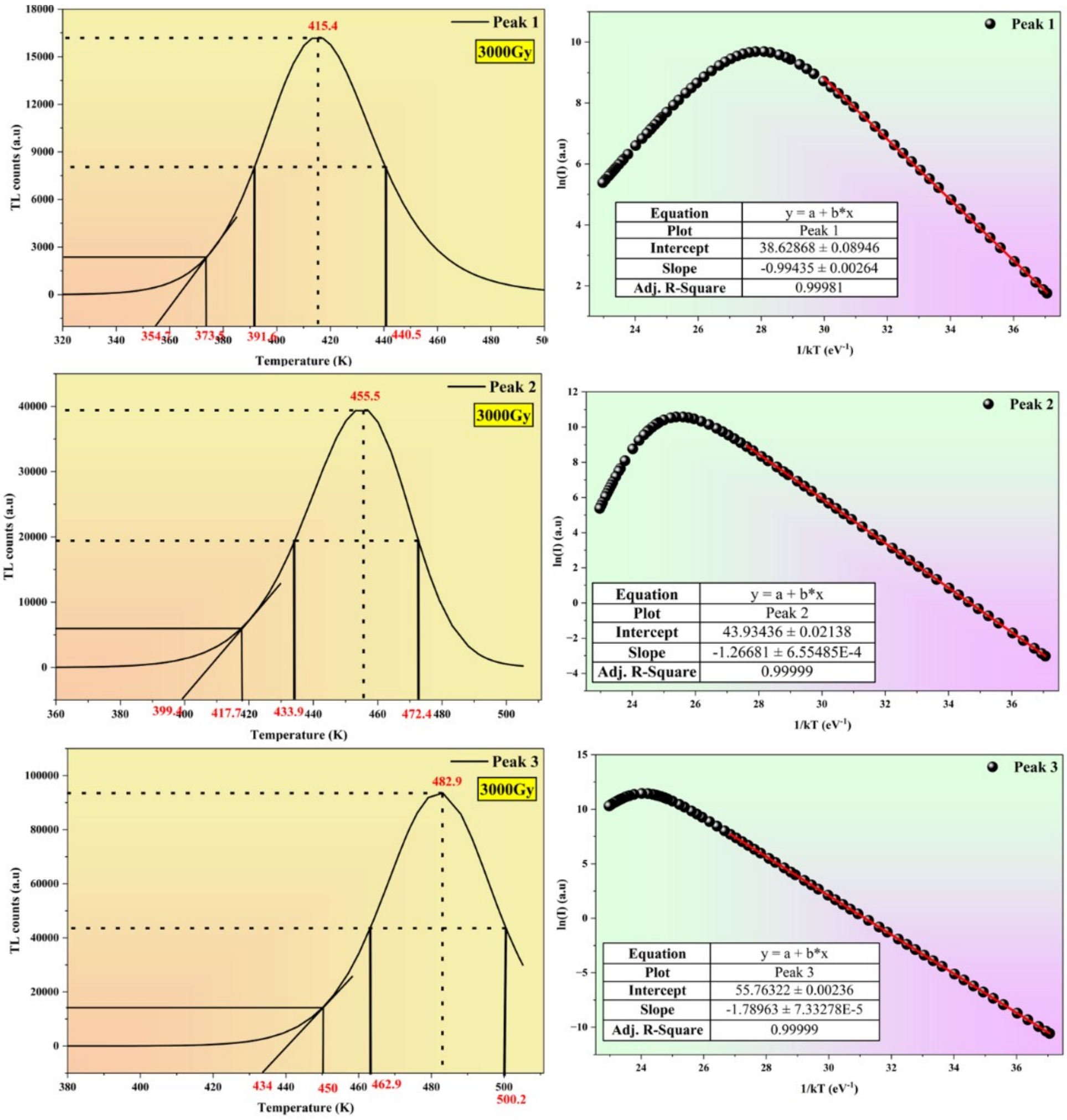

Figure S10. Deconvolution-based TL glow curve study of $Ba_2ZnSi_2O_7$:1 mol% Tb^{3+},1.75 mol% Dy^{3+} at 3000 Gy, with corresponding ln(I) versus 1/kT graph.

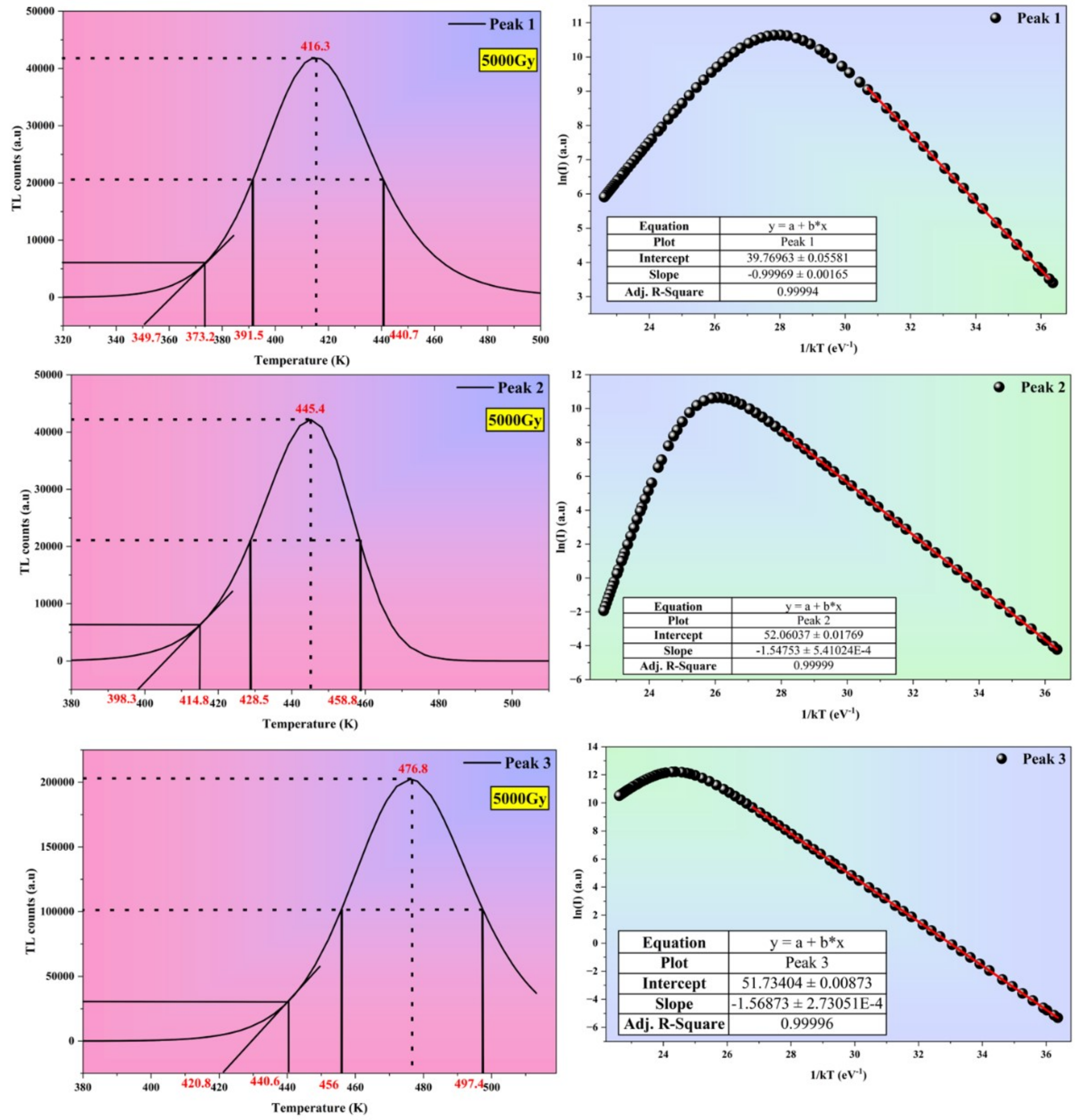

Figure S11. Thermoluminescence glow curve fitting of $Ba_2ZnSi_2O_7$:1 mol% Tb^{3+},1.75 mol% Dy^{3+} at a dose of 5000 Gy, showing individual peaks and ln(I) vs 1/kT plot.

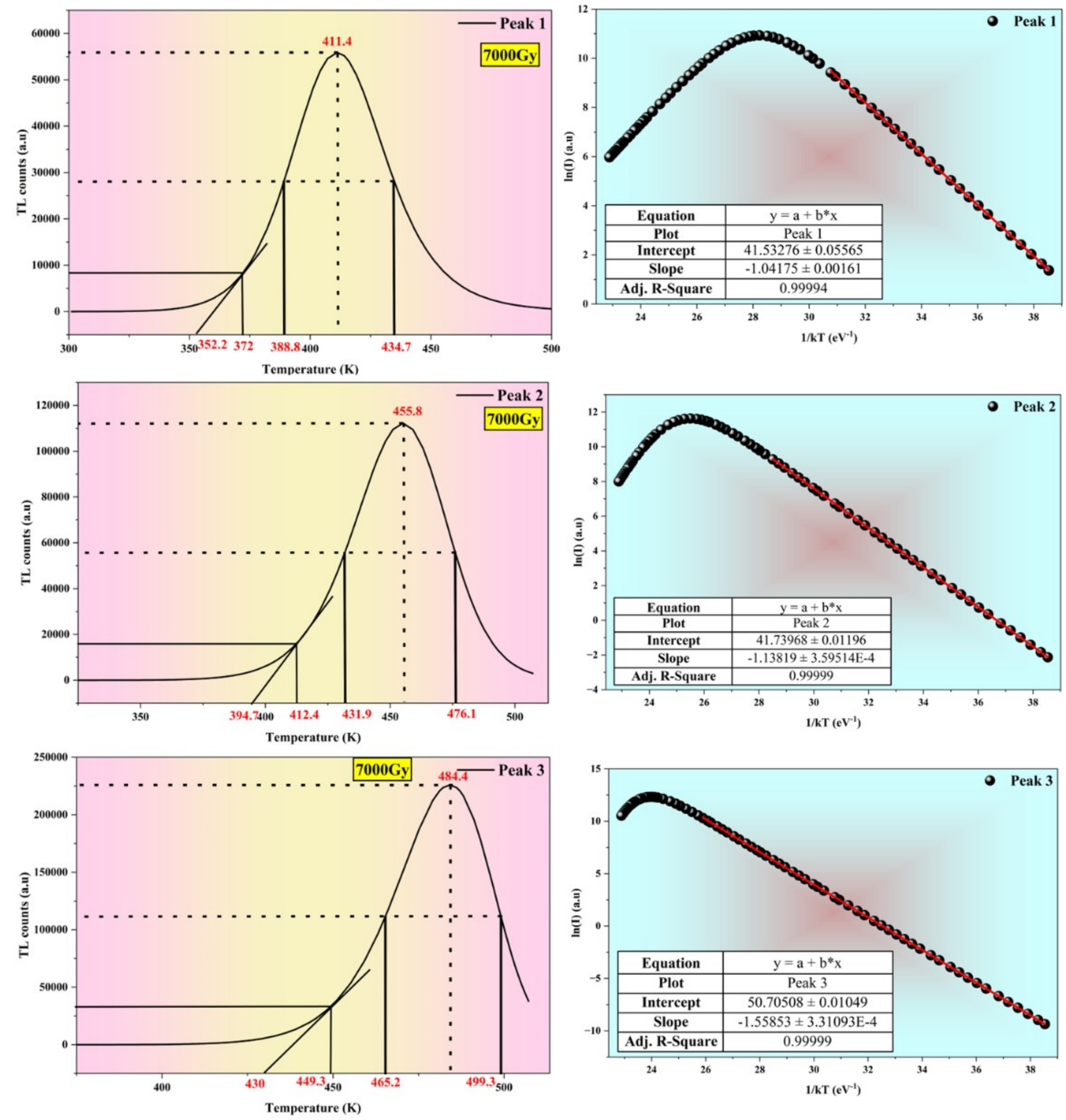

Figure S12. Peak resolved TL glow curve of $Ba_2ZnSi_2O_7$:1 mol% Tb^{3+},1.75 mol% Dy^{3+} phosphor irradiated at 7000 Gy, accompanied by the ln(I) against 1/kT representation.
